# The *Campylobacter jejuni* CiaD effector co-opts the host cell protein IQGAP1 to promote cell entry

**DOI:** 10.1038/s41467-021-21579-5

**Published:** 2021-02-26

**Authors:** Nicholas M. Negretti, Christopher R. Gourley, Prabhat K. Talukdar, Geremy Clair, Courtney M. Klappenbach, Cody J. Lauritsen, Joshua N. Adkins, Michael E. Konkel

**Affiliations:** 1 School of Molecular Biosciences, College of Veterinary Medicine, Washington State University, Pullman, WA USA; 2 Integrative Omics, Pacific Northwest National Laboratory, Richland, WA USA

**Keywords:** Cytoskeleton, Bacteria, Cellular microbiology, Pathogens

## Abstract

*Campylobacter jejuni* is a foodborne pathogen that binds to and invades the epithelial cells lining the human intestinal tract. Maximal invasion of host cells by *C. jejuni* requires cell binding as well as delivery of the Cia proteins (*Campylobacter* invasion antigens) to the host cell cytosol via the flagellum. Here, we show that CiaD binds to the host cell protein IQGAP1 (a Ras GTPase-activating-like protein), thus displacing RacGAP1 from the IQGAP1 complex. This, in turn, leads to the unconstrained activity of the small GTPase Rac1, which is known to have roles in actin reorganization and internalization of *C. jejuni*. Our results represent the identification of a host cell protein targeted by a flagellar secreted effector protein and demonstrate that *C. jejuni*-stimulated Rac signaling is dependent on IQGAP1.

## Abstract (web summary)

The pathogen *Campylobacter jejuni* invades intestinal cells after secreting protein effectors into the host cell cytosol via the flagellum. Here, Negretti et al. show that one of these effectors, CiaD, binds to host protein IQGAP1, thus leading to unconstrained activity of small GTPase Rac1, which modulates actin reorganization and bacterial internalization.

## Introduction

A common strategy for disease-causing microbes is to manipulate the behavior and function of host cells within infected tissues^1^. Bacterial cells hijack a broad array of host cell functions, including blocking immune signaling, promoting fluid secretion, and co-opting cytoskeletal elements to promote their spread and dissemination^2–4^. Importantly, bacterial factors directly target and modulate host cell signaling pathways to redirect cellular processes. Many pathogens have evolved specialized secretion systems to deliver proteins into the cytosol of host cells and rewire the cellular signaling networks in a way that benefits the pathogen^5^.

*Campylobacter jejuni* is among the most common bacterial causes of foodborne illness and diarrhea worldwide due, in part, to its ability to colonize food animals (particularly chickens) as a commensal organism of the intestinal tract^6^. Individuals with campylobacteriosis often develop a fever, headache, abdominal pain, nausea, and diarrhea that is frequently bloody^7^. *C. jejuni* infection is associated with the development of Guillain-Barré syndrome (GBS) and Miller Fisher syndrome, autoimmune disorders affecting the peripheral nervous system^8^. *C. jejuni* is also implicated in the development of irritable bowel disease^9^. *C. jejuni* invasion of intestinal epithelial cells has been observed in natural human cases, nonhuman primate infection studies, and mouse models of infection^10–12^. Furthermore, the presence of blood in the stool of infected individuals is indicative of intestinal cell invasion^13^. These observations support the proposal that human disease is dependent on successful intestinal colonization and subsequent invasion of the epithelial cells lining the intestinal tract.

The results from the past 30 years of work on *C. jejuni* pathogenesis have repeatedly reminded researchers that *C. jejuni* is a unique pathogen. Despite causing similar disease symptoms to other Gram-negative enteric pathogens, the genome of *C. jejuni* is relatively small at only ~1.6 Mbp (~31% GC) compared to that of *Salmonella enterica*, *Yersinia enterocolitica*, and *Shigella flexneri*, which range between 4.6 and 4.8 Mbp (47–52% GC). Moreover, the process of effector protein delivery and the mechanistic basis underpinning the cellular invasion of *C. jejuni* differs from other intestinal pathogens. There is no evidence that *C. jejuni* has a secretion system dedicated to the export of virulence proteins, like the specialized type III secretion system (T3SS) injectisomes of the pathogens listed above. However, *C. jejuni* possesses a bifunctional flagellar export apparatus that secretes structural components of the flagella in addition to virulence proteins. Dedicated T3SS injectisomes and flagellar secretion systems share analogous proteins, and there is evidence that proteins are capable of being secreted from both the dedicated T3SS injectisome and the flagellar system^14–16^. To date, it is known that *C. jejuni* synthesizes at least eight non-flagellar proteins that are secreted from the bacterium in a manner that is dependent on the flagellar T3SS^17–19^. A subset of these proteins, known as the *Campylobacter* invasion antigens (Cia), exert their actions within the cytosol of host cells to manipulate signaling processes^20,21^. Three of these Cia proteins (CiaC, CiaD, and CiaI) have been demonstrated to be delivered to the cytosol of host cells, using a process that is dependent on direct bacterial contact and the presence of a functional flagellar export apparatus^22,23^.

*C. jejuni* possesses a few well-characterized virulence factors that facilitate cellular invasion. For example, the *C. jejuni* CadF and FlpA Fibronectin-binding proteins (FNBPs) facilitate *C. jejuni* attachment to cells, effector protein delivery, and, in part, initiation of the cell signaling that leads to bacterial invasion^24,25^. The *C. jejuni* adhesins promote the formation of a three-component bridge comprised of the FNBPs, fibronectin, and the host cell fibronectin-binding integrin receptors. Signals from the integrins are transmitted through a multiprotein complex known as the focal adhesion, a structure that links the extracellular environment to the actin cytoskeleton, which is central to cellular invasion by *C. jejuni*^26,27^. The signaling events initiated by the *C. jejuni* adhesins are further enhanced by the *C. jejuni* effector proteins CiaC and CiaD to promote maximal *C. jejuni* uptake by host cells^22^. Of these proteins, CiaD (also known as Cj0788c in *C. jejuni* strain NCTC 11168) is most well understood. CiaD has been shown to contribute to cellular invasion in vitro in *C. jejuni* strain F38011 by targeted mutagenesis^28^ and in strain 81–176 by screening a transposon library^29^. Perhaps most importantly, CiaD was demonstrated to contribute to *C. jejuni* disease in a mouse model by investigators blinded to the identities of the infectious isolates^23^. Compared to infection with the *C. jejuni* wild-type strain, mice infected with the *ciaD* deletion mutant have reduced GI tract wall thickening and reduced inflammation of the ileocecocolic lymph node^23^. More recently, the application of a pig ligated loop model also revealed that a *C. jejuni ∆ciaD* mutant does not induce the characteristic robust inflammatory response observed with a *C. jejuni* wild-type strain, as demonstrated by the production of inflammatory cytokines and neutrophil markers^30^. Laboratory studies have shown that CiaD is mechanistically involved in host cell uptake of *C. jejuni* by promoting actin reorganization and membrane ruffling via Erk 1/2 activation^28^. Activated Erk 1/2 phosphorylates (activates) cortactin, which promotes membrane ruffles by binding to the actin remodeling proteins N-WASP and WAVE2^31^. These ruffles are evident in scanning electron micrographs of the infected cells and promote *C. jejuni* uptake.

*Campylobacter* researchers continue to debate the mechanistic basis for *C. jejuni* invasion of epithelial cells, largely because no direct cellular target has been identified for an effector protein. The goal of this study was to identify a host cell target of the *C. jejuni* CiaD effector protein. Previous findings demonstrated that cortactin is activated in a CiaD-dependent manner and that cortactin acts in concert with N-WASP to activate the Arp2/3 complex involved in the initiation of actin polymerization and the reorganization of actin filaments^28^. Therefore, we hypothesized that CiaD interacts with a host cell protein central to actin rearrangement. Additionally, it is known that actin reorganization mediators, including the small GTPases Rac1 and Cdc42, play important roles in the internalization of *C. jejuni*^32^.

In this work, we show that CiaD binds to the host cell protein IQGAP1 to displace RacGAP1 using affinity pull-down experiments coupled with LC–MS/MS and immunoblots, yeast-two hybrid (Y2H), and *C. jejuni*-host cell infection assays. We demonstrate that CiaD promotes *C. jejuni* cell invasion by modifying the IQGAP1 complex to promote signaling through the Rac1 pathway. This represents an important identification of a direct host cell interaction partner for a flagellar effector protein from a bacterial pathogen. This finding demonstrates that *C. jejuni* invasion of cells is a pathogen driven process that is dependent on cell binding and effector delivery (a binding and effector mechanism).

## Results

### Identification of eukaryotic targets of CiaD

It is known that the delivery of CiaD from *C. jejuni* to the cytosol of host cells is necessary for cellular invasion^23^. The purpose of this study was to determine the host cell target protein of CiaD, which is 163 amino acids long with a calculated molecular mass of 19.4 kDa. There are no apparent conserved domains in CiaD that are detected by the NCBI Conserved Domain Search^33^, the InterPro protein sequence analysis and classification system^34^, or when searching Pfam^35^. Although there are no conserved domains, previous studies have found that CiaD contains a Mitogen-activated protein kinase docking (MKD) motif that promotes cellular invasion^23,36^. Due to the presence of the MKD motif, and the phenotypes of the ∆*ciaD* mutant, we focused our efforts on identifying CiaD-interacting proteins that were related to the MAPK pathway.

**Fig. 1:**
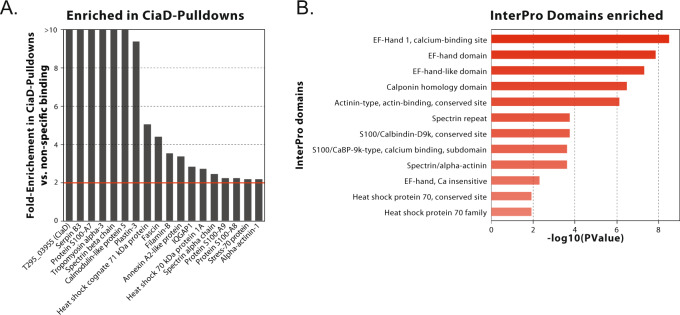
Analysis of CiaD-host cell protein pull-down by LC–MS/MS indicates potential binding partners. **A** CiaD and 17 human proteins, including IQGAP1, were found to be at least 2-fold more abundant in the pull-down experiments compared to similar experiments performed without CiaD. **B** Protein domain enrichment analysis was performed using DAVID on the list of proteins that were identified by the pull-down. Enriched InterPro protein domains that are involved in calcium signaling and actin binding were identified. The *x*-axis indicates the *p*-value from a one-tailed Fisher’s exact test.

We initially used purified CiaD to pull-down proteins from lysates prepared from cultured human INT 407 epithelial cells. The pull-down experiments were performed with a glutathione-S-transferase (GST)-tagged CiaD protein, and the resulting protein complexes were subjected to LC–MS/MS for identification using a spectral counting strategy with the search engine MS-GF+ . The spectral counts were then normalized by the total number of spectra obtained for each assay. The cellular proteins from the CiaD pull-down (*n* = 2) were compared to the cellular proteins pulled down in the absence of recombinant CiaD (*n* = 1) to identify those proteins that bound in a CiaD-dependent manner to the GST affinity column. By comparing the normalized spectral counts for the proteins present in the pull-down experiments to the proteins non-specifically bound to the affinity column, we found that CiaD and 17 human proteins were enriched at least 2-fold in the pull-downs (Fig. 1A and Supplementary Data 1). While these proteins had different levels of enrichment, it was not clear whether this was a result of the number of copies in the protein complex, affinity of the interaction, or expression level in the original sample. Therefore, we considered all 17 candidate proteins with equal likelihood.

Functional enrichment analysis of the 17 human proteins was performed using DAVID^37^ (Fig. 1B). Among the enriched InterPro domains were “Spectrin/alpha-actinin,” “Calponin homology domain,” and “Actinin-type/actin-binding/conserved site.” The latter domain binds to F-actin and was first described in α-actinin, although it is also found in many other actin-binding proteins^38^. The “Spectrin/alpha-actinin” domain forms a three-helix bundle that is commonly found in proteins involved with cytoskeletal structure^39^. The “Calponin homology domains” are found in proteins that link cellular signaling processes to the actin cytoskeleton^40^. The enrichment of actin-binding domains is consistent with the hypothesis that CiaD modifies the activity of the actin cytoskeleton, as the major phenotypes of a ∆*ciaD* mutant are the lack of actin-driven membrane ruffles resulting in reduced *C. jejuni* cellular invasion^28^. Among the cytoskeleton related proteins identified in the pull-down were IQGAP1, which is a scaffolding protein that coordinates actin reorganization, Filamin A and B, which connect cell membrane constituents to the actin cytoskeleton, and Fascin, which organizes filamentous actin into parallel bundles.

A commercial yeast-two hybrid assay (Y2H) was used to screen for direct protein-protein interactions between CiaD and host cell targets (Supplementary Data 2). The CiaD protein was fused to the activation domain of LexA and used as bait to screen a human placenta cDNA library because this library represents a wide variety of transcripts^41,42^. The screen resulted in three high confidence hits (prey proteins): dynein light chain roadblock-type 1 (DYNLRB1), which is an accessory protein of the dynein 1 complex that participates in TGFβ signaling^43^; nuclear receptor-binding protein (NRBP1), which has a role in trafficking between the endoplasmic reticulum and Golgi apparatus; and the Ras GTPase-activating-like protein IQGAP1, which is a scaffolding protein that regulates the actin cytoskeleton. The protein IQGAP1 was identified in both the commercial Y2H and LC–MS/MS screens.

Amino acid alignments between the three potential CiaD targets showed no significant sequence similarity (alignment in Supplementary Fig. 1). Therefore, we examined the region targeted by CiaD within the DYNLRB1, NRBP1, and IQGAP1 proteins. The full-length DYNLRB1 protein was identified in the screen, so binding may occur anywhere in the protein. However, DYNLRB1, at 96 aa, does not contain any specific conserved protein domains. Similarly, NRBP1, for which the CiaD target region was localized between amino acids 457 to 523, does not contain any known protein domains. For IQGAP1, the interacting fragment was localized to the Ras GTPase-activating domain (GRD domain) located between amino acids 964 and 1365. This domain is known to play a role in modulating the activity of Ras GTPases, including Rac1 and Cdc42, which are the critical mediators of actin reorganization.

Because IQGAP1 was the only protein identified in the screening experiments (LC–MS/MS and Y2H) that has a role in the Erk 1/2 signaling pathway and actin reorganization, it was considered a top candidate. The GRD domain of IQGAP1, which is the region that is predicted to interact with CiaD, regulates the activities of the Rac1 and Cdc42 GTPases involved in actin reorganization. Therefore, we focused our efforts on the characterization of the CiaD-IQGAP1 interaction.

### Interactions with IQGAP1

We hypothesized that IQGAP1 represents a genuine cellular target of CiaD based on three findings: (1) IQGAP1 was the only protein identified from both the LC–MS/MS screen and the Y2H screen; (2) cell biologists have demonstrated that IQGAP1 is involved in the activation of the Erk 1/2 signaling pathway and actin reorganization; (3) cellular proteins, which are known to interact with IQGAP1, have been demonstrated to be involved in *C. jejuni* internalization of host cells, including the Rho GTPases Rac1 and Cdc42. More specifically, Rac1 and Cdc42 behave as “molecular switches” to promote membrane ruffling when in the active (GTP-bound) state. In fact, IQGAP1, which is a large (189.3 kDa) scaffolding protein, gets its name from the presence of several IQ domains and a ‘GTPase-activating protein-related’ (GAP) domain (Supplementary Fig. 1). Despite the name of this domain, experimental evidence indicates that the rate of GTP hydrolysis by Cdc42 does not increase when bound to the GAP domain. The current evidence suggests that IQGAP1 may stabilize the active GTP bound form of Cdc42^44^.

**Fig. 2:**
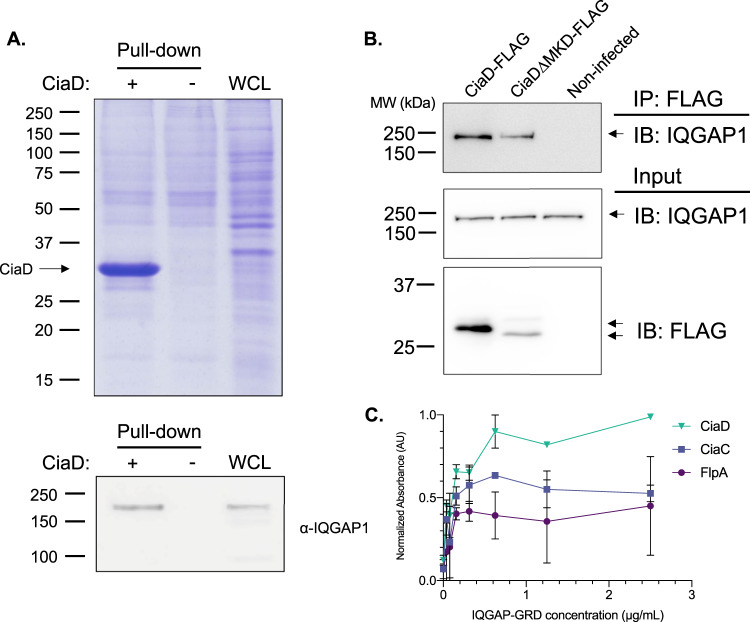
CiaD interacts with IQGAP1. **A**, upper Recombinant His6-CiaD was produced and purified from *E. coli* and then mixed with a whole-cell lysate (WCL) prepared from INT 407 cells. The His6-CiaD was then pulled down using Talon affinity resin. The protein mixtures were separated by SDS-PAGE and stained with Coomassie Brilliant Blue R-250, where recombinant CiaD is clearly visible. **A**, lower The sample was tested for the presence of IQGAP1 by immunoblot, and it was observed that IQGAP1 pulls down in the presence of CiaD (+) but not in the absence of CiaD (−). IQGAP1 is also present in the INT 407 WCL used for the pull-down input. Molecular weights are indicated in kDa. **B** Anti-FLAG immunoprecipitations (IPs) were performed with INT 407 cells that were infected with a *C. jejuni* F38011 ∆*ciaD* mutant harboring either the CiaD-FLAG (CiaD) plasmid or the CiaD∆MKD-FLAG (CiaD∆MKD) plasmid. These constructs express the CiaD proteins from a constitutive promoter. The CiaD∆MKD-FLAG construct is missing the Mitogen-activated protein kinase docking motif (MKD) within CiaD. IPs were probed with an antibody for IQGAP1. It was found that CiaD-FLAG was delivered by *C. jejuni* and associates with IQGAP1. The inputs for the FLAG IPs were also probed for IQGAP1 and FLAG to determine initial protein abundance. Arrows indicate expected molecular weight for each blot. Molecular weights are indicated in kDa. **C** An ELISA was performed to determine the binding between the recombinant GRD domain from IQGAP1 (IQGAP1-GRD), and recombinant *C. jejuni* proteins CiaD, CiaC and FlpA (*n* = 2 independent samples). Binding between the *C. jejuni* proteins was assessed against increasing concentrations of IQGAP1-GRD. CiaD showed the greatest binding potential. Values are shown as normalized absorbance that has been scaled from zero (minimum assay value) to one (maximum assay value). Error bars represent the standard error of the mean.

To confirm the CiaD-IQGAP1 interaction using a third method, a targeted pull-down assay was performed coupled with immunoblot analysis. Pull-down experiments were performed by mixing purified recombinant 6His tagged CiaD with cell lysates from INT 407 cells and then pulling down the resulting protein complexes with TALON affinity resin, which binds to the tagged CiaD. Samples were tested for the presence of IQGAP1 using an immunoblot. In the samples that had the CiaD protein bound to the TALON affinity resin, IQGAP1 was pulled down; IQGAP1 was not identified in the pull-down experiments where CiaD was not added to the reaction mixture (negative control) (Fig. 2A). In this targeted assay, we observed that CiaD bound to IQGAP1, which is in agreement with the data from the non-targeted LC–MS/MS and Y2H screens. Furthermore, we found that IQGAP1 is not pulled down with other *C. jejuni* proteins, including CiaC and FlpA, indicating a specific interaction with CiaD (Supplementary Fig. 2).

To determine if CiaD was specifically binding to the domain identified in the Y2H, the GRD domain of IQGAP1 was expressed and purified from *E. coli*. Using an ELISA, it was found that CiaD was able to bind to the GRD domain of IQGAP1, and that this saturable interaction was stronger than other *C. jejuni* proteins, including CiaC and FlpA (Fig. 2C). This finding supports the conclusion that CiaD directly interacts with IQGAP1 and that this interaction occurs at the GRD domain.

Based on the findings from the pull-down experiments coupled with LC–MS/MS, Y2H, and directed pull-down experiments, CiaD binds to IQGAP1. However, the context of cellular infection is different from protein:protein interactions in vitro. The purpose of the infection experiments was to test whether the CiaD protein was binding to IQGAP1 during cellular infection. In addition, if we observed binding of the *C. jejuni* CiaD protein to the IQGAP1 protein, this would indicate that the CiaD protein was successfully translocated across the host cell plasma membrane to interact with a cytosolic protein. Therefore, we infected cells with a *C. jejuni* ∆*ciaD* mutant harboring a plasmid with either the CiaD-FLAG or CiaD∆MKD-FLAG insert driven by a constitutive promoter. After *C. jejuni* infection, the cells were lysed, and immunoprecipitations were performed with an anti-FLAG affinity gel. We found that when the cells were infected with *C. jejuni* expressing CiaD-FLAG or CiaD∆MKD-FLAG, IQGAP1 was immunoprecipitated using the anti-FLAG affinity gel (Fig. 2B). This finding indicates that during the course of a *C. jejuni-*host cell infection, the CiaD protein is delivered to the cytosol of the host cells and interacts with IQGAP1. In addition, the data indicates that the interaction of CiaD with IQGAP1 is independent of its MKD domain. The MKD domain of CiaD is necessary to promote Erk 1/2 activation and is involved in cellular invasion by *C. jejuni*^23^. Importantly, this finding shows that the native *C. jejuni* protein interacts with IQGAP1 during the course of normal cellular infection.

### Role of IQGAP1 for cellular invasion

It is well established that CiaD facilitates invasion into host cells, as the deletion of *ciaD* reduces cellular invasion in multiple strains of *C. jejuni*^23,29^. Because we have found that CiaD interacts with IQGAP1, we predicted that IQGAP1 is required for maximal *C. jejuni* cell invasion. Using lentiviral delivered shRNA, we generated clonal INT 407 cell lines that synthesized reduced levels of IQGAP1. Two independent shRNAs were used, which had different targets on the IQGAP1 mRNA. The shRNA labeled 928 targets the coding sequence of IQGAP1, while the shRNA labeled 930 targets the 3′-UTR. Both of the shRNA treatments resulted in non-detectable levels of IQGAP1 in the two clonal lines tested (Supplementary Fig. 3). We then measured the ability of *C. jejuni* to adhere to the INT 407 cells and the ability of *C. jejuni* to invade the cells using the gentamicin protection assay. In all four clonal cell lines tested, there was no reduction in the adherence of *C. jejuni* compared to cells expressing a non-targeting shRNA. However, there was a statistically significant reduction in invasion when compared to a cell line that was expressing a non-targeting shRNA (*p* < 0.05 as determined by ANOVA followed by Sidak’s test). The average reduction in cell invasion was approximately 50% (or 0.5 log). There was no significant difference in the invasion between the different clones that were targeting two different portions of the mRNA. Given the similar results observed with each of the two independent INT 407 IQGAP1 knockdown cell lines (each generated with the two different IQGAP1 shRNA constructs), we concluded that the reduction in cellular invasion by *C. jejuni* was specific to the reduction of IQGAP1 levels.

**Fig. 3:**
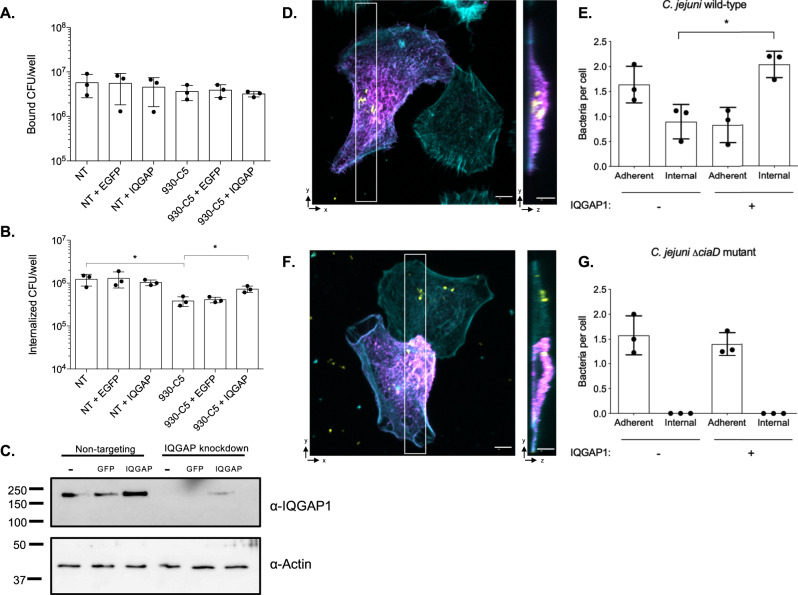
IQGAP1 is required for cellular invasion by *C. jejuni*. Stable INT 407 cell lines expressing a non-targeting (NT) shRNA or an IQGAP1-targeting shRNA (930-C5) were generated. Rescue experiments were performed by transfecting the stable clones with the pCMV-EGFP-Myc (EGFP) or pCMV-IQGAP1-Myc (IQGAP1) plasmids. Cellular adherence and internalization of the *C. jejuni* wild-type strain were measured. **A** Knockdown or rescue of IQGAP1 does not alter the cellular adherence of *C. jejuni* to any of the cell types tested (*n* = 3 independent samples). **B** Knockdown of IQGAP1 reduces *C. jejuni* internalization while expressing IQGAP1-Myc improves invasion. In panels **A** and **B**, error bars represent the standard deviation; differences were compared using an ANOVA on log-transformed counts followed by a two-tailed Sidak’s test (*p < 0.05) (*n* = 3 independent samples). Comparing NT to 930-C5, *p* = 0.0007. Comparing 930-C5 to 930-C5 + IQGAP1, *p* = 0.0472. **C** The amount of IQGAP1 in the cell lines was determined by immunoblot. Actin was used as a loading control for the cell lysates. Molecular weights are indicated in kDa. **D** To test if the mosaic nature of transient transfection was responsible for the incomplete restoration of invasion in the IQGAP1 knockdown cells that were transfected with IQGAP1, we used microscopy to assess the internalization of *C. jejuni* into cells. IQGAP1 knockdown cells were transfected with pCMV-IQGAP1-Myc, infected with *C. jejuni* for 10 h, then fixed and stained for *C. jejuni* (yellow), actin (cyan), and with an anti- Myc antibody (magenta). A z-section was constructed from the section in the white box (right). From the z-section, it is apparent that the *C. jejuni* associated with the cells expressing IQGAP1 are internalized. **E** Three biological replicates were imaged, and more than 15 cells were analyzed for each condition (IQGAP1 expressing and non-expressing). We found that the number of internalized *C. jejuni* was higher in the cells expressing IQGAP1. White bars represent 5 µm. Comparing internalized bacteria in IQGAP1 negative to IQGAP1-positive cells, *p* = 0.0061. **F** Cells were infected with a *C. jejuni* Δ*ciaD* mutant and imaged. From the z-section, it is clear that the *C. jejuni* Δ*ciaD* mutant does not invade cells, regardless of whether IQGAP1 is present. White bars represent 5 µm. **G** From three biological replicates, greater than 15 cells were analyzed, and the number of internal and external *C. jejuni* were determined. No internalized bacteria were observed in cells infected with the *C. jejuni* Δ*ciaD* mutant. In panels **E** and **G**, each dot represents a biological replicate, the column represents the mean, error bars represent standard deviation, and significant differences were assessed using a two-tailed Student’s *t*-test (*p < 0.05).

To confirm that the knockdown of IQGAP1 was specifically responsible for the reduction in cellular invasion, the expression of IQGAP1 was restored in a knockdown cell line (named 930-C5). Because the IQGAP1 expression vector does not contain the IQGAP1 3′ UTR that is present on the native mRNA, no modification of the complementation vector was needed to avoid the effects of the shRNA. Cellular invasion was increased in the knockdown cell line expressing IQGAP1-Myc but was not changed in the cells expressing the vector that contained EGFP in place of IQGAP1, as judged by the gentamicin protection assay (Fig. 3). While expression of IQGAP1-Myc in the knockdown cells significantly increased invasion compared to the knockdown cells alone, it was not completely restored to the level of cells expressing the non-targeting shRNA. The partial restoration of invasion is consistent with the fact that transient transfection is not completely efficient and that the level of IQGAP1 expression was not the same as in wild-type cells. This finding is supported by immunoblot, where the amount of IQGAP1 protein was lower than in the wild-type cells (Fig. 3C). Finally, the invasion of *C. jejuni* into the cell line producing the non-targeting shRNA was not affected when IQGAP1 was overexpressed by transfection with the pCMV-IQGAP1-Myc vector (Fig. 3D). This indicates that a biologically relevant amount of IQGAP1 is necessary for *C. jejuni* cellular invasion and that increased levels of IQGAP1 within a cell do not enhance cell invasion. Most importantly, these findings demonstrate that IQGAP1 has a functional role in *C. jejuni* invasion.

While the gentamicin protection assay is the most widely used method to assess the efficiency of bacterial internalization, it only provides global information about the number of bacteria internalized by a population of cells and does not address bacterial internalization into a single cell. To test if the varying levels of IQGAP1 expression were responsible for the partial complementation of cellular invasion by *C. jejuni*, we used confocal microscopy to assess bacterial internalization in the 930-C5 cell line that was transfected with the IQGAP1-Myc expressing plasmid. The *C. jejuni* infected cells were immunostained for actin, *C. jejuni*, and with an anti-Myc antibody to detect IQGAP1 expression (Fig. 3D–G). Confocal examination of the cells revealed that the number of *C. jejuni* internalized was significantly greater in cells expressing IQGAP1 than in cells lacking IQGAP1. Consistent with the gentamicin protection assay, the number of internalized *C. jejuni* was reduced by approximately 50% in cells lacking IQGAP1. Importantly, the total number of cell-associated *C. jejuni* (adherent and internalized) was similar for the IQGAP1-positive and negative cells (no significant difference, *p* = 0.415 by Student’s *t*-test). Consistent with previous observations, the *C. jejuni* ∆*ciaD* mutant did not invade cells regardless of the presence of IQGAP1 (Fig. 3F, G). Taken together, these findings demonstrate that IQGAP1 is necessary for *C. jejuni* to maximally invade cells at high efficiency.

### Signaling events associated with cellular invasion

**Fig. 4:**
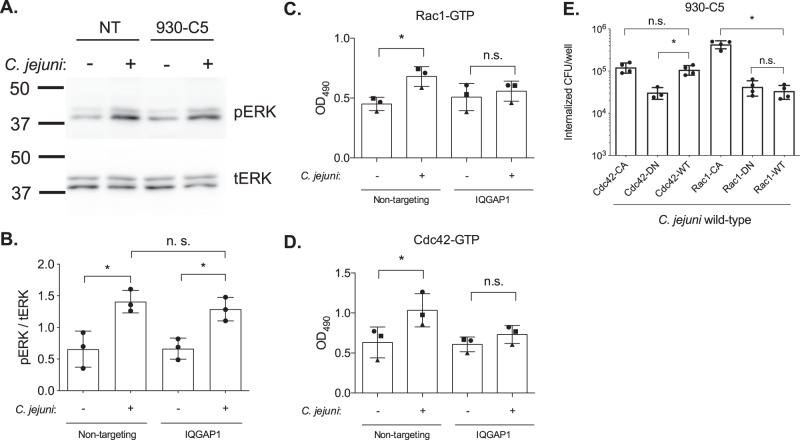
*C. jejuni* mediated ERK activation occurs upstream of IQGAP1, while both Rac1 and Cdc42 activation is dependent on IQGAP1. **A** Human INT 407 cells expressing a non-targeting shRNA (NT) or shRNA targeting the 3′-UTR of IQGAP1 (930-C5) were serum-starved for 4 h, then infected with *C. jejuni* for 45 min. Samples were collected and analyzed by immunoblot for phosphorylated ERK and total ERK in parallel. A representative immunoblot is shown. Molecular weights are indicated in kDa. **B** The ratio between the intensity of the phospho-ERK and total-ERK bands were quantified from three independent biological replicates and plotted. Comparing pERK/tERK in non-targeting shRNA cells with and without *C. jejuni p* = 0.0069, and in IQGAP1 knockdown cells *p* = 0.0193. **C** We observed that in cells expressing non-targeting shRNA there was a robust activation of Rac1 in the presence of *C. jejuni*, and there was not a significant amount of activation of Rac1 in the IQGAP1 knockdown cells. Each symbol represents the results from a biological replicate (*n* = 3 independent samples). Comparing Rac1-GTP levels in non-targeting shRNA cells with and without *C. jejuni, p* = 0.0339, and in IQGAP1 knockdown cells *p* = 0.8751. **D** A similar trend was observed for the amount of Cdc42 activation, where knockdown of IQGAP1 inhibited *C. jejuni*-induced Cdc42 activation. Each symbol represents the results from a biological replicate (*n* = 3 independent samples). Comparing Cdc42-GTP levels in non-targeting shRNA cells with and without *C. jejuni, p* = 0.0435, and in IQGAP1 knockdown cells *p* = 0.7488. **E** IQGAP1 knockdown cells (930-C5) were transfected with constitutive active (CA), dominant-negative (DN) or wild-type (WT) Cdc42 and Rac1, and the amount of internalized *C. jejuni* was measured in each condition using a gentamicin protection assay (*n* = 4 independent samples). The following comparisons were made on log-transformed counts: Cdc42-CA to Cdc42-WT (*p* = 0.9650), Cdc42-DN to Cdc42-WT (*p* = 0.0002), Rac1-CA to Rac1-WT (*p* = <0.0001), Rac1-DN to Rac1-WT (*p* = 0.7861). For all charts, bars represent the mean and error bars represent the standard deviation; significant differences were determined by ANOVA followed by a two-tailed Sidak’s multiple comparisons test (*p < 0.05).

One of the most striking phenotypes of a *C. jejuni* ∆*ciaD* mutant is the lack of activation (phosphorylation) of the MAPK signaling cascade, as evidenced by the lack of Erk 1/2 phosphorylation^23^. Given that IQGAP1 acts as a scaffold for Erk 1/2, we tested if IQGAP1 was facilitating the CiaD mediated activation of ERK signaling. Cells expressing non-targeting shRNA and IQGAP1 knockdown cells were serum-starved for 4 h and infected with *C. jejuni*. As reported previously, Erk 1/2 is activated by infection with a *C. jejuni* wild-type strain when compared to non-infected cells. However, the amount of Erk 1/2 activation by *C. jejuni* was not reduced when IQGAP1 was knocked down (Fig. 4A, B). This is consistent with the finding that the MKD domain of CiaD is not involved in IQGAP1 binding and suggests that the CiaD protein activates ERK signaling upstream or independently of IQGAP1.

As ERK signaling was not impaired in the IQGAP1 knockdown cells, we wanted to determine the functional reason for the reduction in invasion in the IQGAP1 knockdown cells. The small Rho GTPases Rac1 and Cdc42 are important in invasion and actin reorganization, so we tested the amount of Rac1 and Cdc42 activation in wild-type and IQGAP1 knockdown cells. Rac1 and Cdc42 G-LISA kits were used to determine the amount of active (GTP bound) Rac1 and Cdc42 present in the cells after infection. We found that there was nearly no activation of either Rac1 and Cdc42 in the IQGAP1 knockdown cells, while there was robust activation of both Rac1 and Cdc42 in the cells expressing the non-targeting shRNA during infection (Fig. 4C, D). This finding indicates that IQGAP1 is facilitating actin reorganization by signaling through both Rac1 and Cdc42 during the process of *C. jejuni* cellular invasion. This also supports the conclusion that IQGAP1 mediates the Rho GTPase signaling that participates in *C. jejuni* invasion.

To test if the signaling through Rac1 and Cdc42 is sufficient to facilitate invasion, we expressed constitutive-active forms of Rac1 and Cdc42 in the IQGAP1 knockdown cells (Fig. 4E). In the IQGAP1 knockdown cells, we found that expressing the constitutive-active (CA) Cdc42 construct did not increase invasion above wild-type (WT) Cdc42, while the dominant-negative (DN) reduced invasion. In contrast, we found that the Rac1-CA increased invasion above Rac1-WT, and the Rac1-DN did not reduce invasion. Relevant to this finding is that cell viability and transfection efficiency varied by less than 7% among all of the samples (Supplementary Fig. 4). This suggests that while both Rac1 and Cdc42 activation are impaired in the IQGAP1 knockdown cells, Rac1 is primarily responsible for facilitating *C. jejuni* invasion through IQGAP1 signaling.

**Fig. 5:**
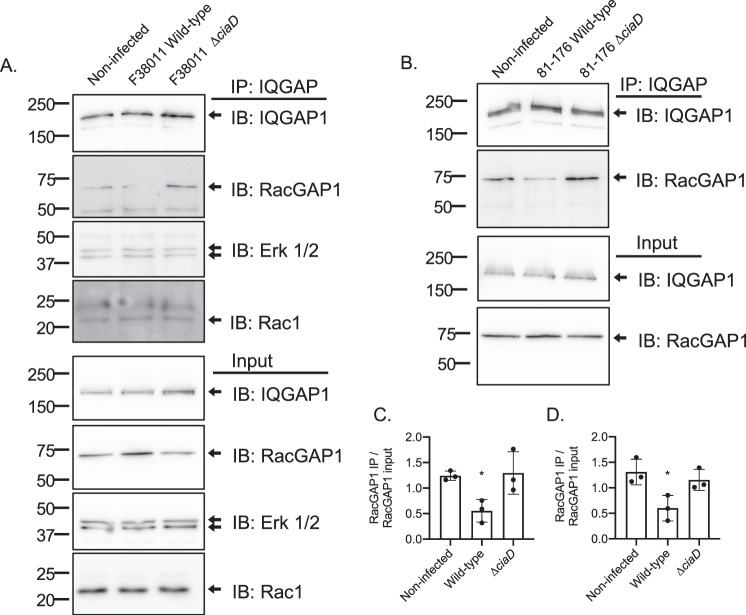
The CiaD effector protein binds to IQGAP1 to exclude RacGAP1. **A** IQGAP1 immunoprecipitations (IPs) were performed with INT 407 cells that were non-infected, infected with the *C. jejuni* F38011 wild-type strain, or infected with the *C. jejuni* ∆*ciaD* mutant. Pull-downs were probed by immunoblot (IB) with antibodies for IQGAP1 and RacGAP1. The amount of RacGAP1 in the IQGAP1 complex was reduced during infection with the *C. jejuni* wild-type strain but not with the ∆*ciaD* mutant. The amount of Erk 1/2 and Rac1 was not changed. The protein mixture used as the input for the IQGAP1 IPs were also probed for IQGAP1, RacGAP1, Erk 1/2, and Rac1 to determine initial protein abundance. Molecular weights are indicated in kDa. **B** Similar IQGAP1 IPs were performed with INT 407 cells that were non-infected, and infected with the *C. jejuni* 81–176 wild-type strain. Quantitation of the amount of RacGAP1 in three biologically independent IP experiments indicated that the amount of RacGAP1 in the IQGAP1 complex was reduced approximately 50% during infection with the **C** *C. jejuni* F38011 wild-type strain and the (*p = 0.0186) Molecular weights are indicated in kDa. **D** *C. jejuni* 81–176 wild-type strain. Bars represent the mean and error bars represent the standard deviation; significant differences from the non-infected samples were determined by ANOVA followed by a two-tailed Dunnet’s multiple comparisons test (*p = 0.0401).

Although we have demonstrated that CiaD binds to IQGAP1, this observation, in itself, does not explain why this protein-protein interaction is necessary for *C. jejuni* invasion. To explore the mechanistic basis for CiaD function (promotion of actin-based invasion by *C. jejuni*), we performed IQGAP1 immunoprecipitation experiments to determine the composition of the IQGAP1 complex. We chose to determine the abundance of three ‘actin regulator proteins’ based on the finding that Rac1-CA enhanced the invasion of the *C. jejuni* in IQGAP1 knockdown cells versus cells transfected with Rac1-WT construct and because the Cdc42-CA construct did not rescue cell invasion. Specifically, we probed for three of the components of the IQGAP1 complex (Rac1, Erk 1/2, and RacGAP1). Rac1 and Erk 1/2 are mediators of *C. jejuni* dependent actin reorganization, and RacGAP1 is a mediator of Rac1 activity that associates with IQGAP1 to constrain Rac1 activity^45^. Immunoprecipitations were performed on lysates from noninfected cells, cells infected with the *C. jejuni* wild-type strain, and cells infected with the ∆*ciaD* mutant. We did not observe a difference in the abundance of Rac1 and Erk 1/2 in the IQGAP1 complex (Fig. 5A). However, we found that the association of RacGAP1 with IQGAP1 was reduced approximately 50% upon infection with a *C. jejuni* wild-type strain but not upon infection with a ∆*ciaD* mutant (*p* < 0.05). This result was consistent with both the *C. jejuni* F38011 and *C. jejuni* 81–176 strains, demonstrating that at least two pathogenic strains that cause bloody diarrhea in infected individuals utilize the same mechanism for cell invasion (Fig. 5A, B). In summary, this finding indicates that the presence of CiaD excludes RacGAP1 from the IQGAP1 complex to potentially increase the local Rac1 activity.

## Discussion

**Fig. 6:**
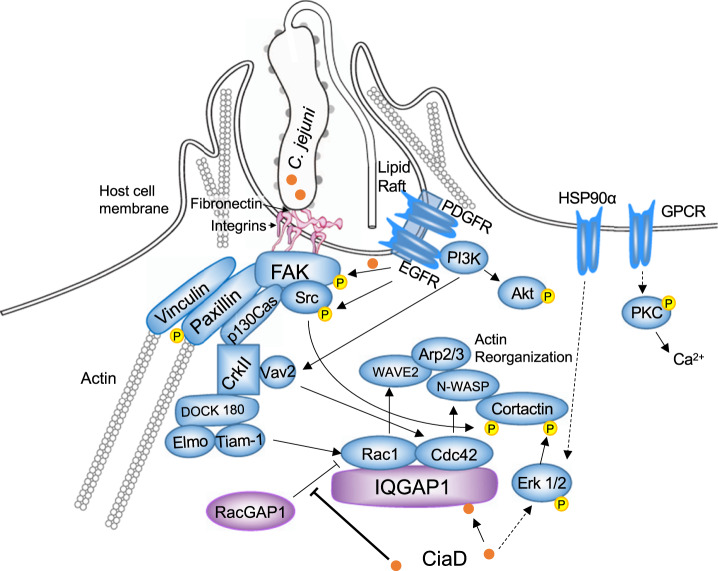
Proposed model of the role of the *C. jejuni* effector protein CiaD in the process of internalization based on experimental results and other published literature. The first step in cellular invasion is the adherence of *C. jejuni* to the host fibronectin via the CadF and FlpA adhesins (gray dots on the *C. jejuni*). Next, the *C. jejuni* flagellar-dependent effector proteins, including CiaD (orange circles), are delivered to the cytosol of the host cell. The combined action of bacterial adherence and the secreted effector proteins triggers activation (phosphorylation) of the focal adhesion components FAK, Src, and paxillin, which recruits vinculin. Focal adhesion signaling is integrated into actin remodeling signaling through the proteins p130Cas, CrkII, DOCK 180, Elmo, and Rac1. Cytosolic CiaD facilitates the activation of the Erk 1/2 signaling pathway, which results in the phosphorylation of cortactin. IQGAP1 serves as a binding partner of CiaD, and it facilitates the activation of Rac1 and Cdc42, further promoting actin reorganization and bacterial uptake through the proteins WAVE2, N-WASP, and Arp2/3. CiaD promotes Rac1 activation by excluding RacGAP1 from the IQGAP1 complex, preventing the deactivation of Rac1. Other literature has also provided support for the roles of EGFR, PDGFR, PI3K, Akt, HSP90α, GPCRs, PKC, Vav2, and Tiam-1 in coordinating bacterial uptake. Purple indicates the findings from this study.

Most of the knowledge about host cell components involved in *C. jejuni* internalization comes from knockdown or inhibitor studies^23,27,28,32,46–50^. These studies have revealed that *C. jejuni* targets focal adhesions, a large multiprotein complex in host cells that links the extracellular matrix to the actin cytoskeleton. The first step in *C. jejuni-*induced cell invasion is adherence to the cell-associated fibronectin via the CadF and FlpA FNBPs^47,51^, which initiate outside-in signaling through the focal adhesions as evidenced by paxillin phosphorylation^27^. Direct bacteria-host cell contact further permits the translocation of the flagellar-dependent Cia effector proteins, including CiaD, into the host cell cytosol. Cytosolic CiaD facilitates the activation of the Erk 1/2 signaling pathway and cellular invasion in a mechanism dependent on its MKD motif^23^. The presence of CiaD results in the phosphorylation and activation of cortactin, an actin remodeling protein^28^. Using both non-targeted assays (affinity pull-down experiments coupled with LC–MS/MS and Y2H), as well as targeted pull-down experiments coupled with immunoblots, we identified IQGAP1 as a binding partner of CiaD. We further found that IQGAP1 is required for the maximal internalization of *C. jejuni* through its interaction with Rac1 and Cdc42. Importantly, we also found that CiaD keeps Rac1 active by displacing RacGAP1 from the IQGAP1 complex and that CiaD has an IQGAP1 independent mechanism to activate the Erk 1/2 signaling pathway. These findings point to a mechanism whereby IQGAP1 is downstream in the signaling cascade that promotes *C. jejuni* uptake, with additional upstream or parallel pathways also acting to facilitate invasion. We show a model of *C. jejuni* internalization, where CiaD binds to IQGAP1, increasing Rac1 activation by displacing RacGAP1, and promoting *C. jejuni* internalization in Fig. 6. IQGAP1 plays a central role in integrating the signals from focal adhesions to actin through Rac1 and Cdc42. While previous studies have sought to define the host proteins necessary for *C. jejuni* internalization, these results now demonstrate that a *C. jejuni* effector protein binds to a host cell component and alters its inherent activity. In addition, we now implicate both IQGAP1 and RacGAP1 in *C. jejuni* internalization. Taken together, the finding that CiaD displaces RacGAP1 from IQGAP1 to promote Rac1 activity supports the proposal that *C. jejuni* internalization is a bacterial driven process, where the bacterium utilizes a binding and effector mechanism for cell invasion.

The terms ‘type III secretion system’ (T3SS) and ‘injectisome’ have been used to describe related sets of contact-dependent secretions systems^52^. The best-understood T3SSs are present in human pathogens that have the most tools for genetic manipulation, including *E. coli*, *Yersinia, Pseudomonas*, and *Salmonella*^53–56^. Among bacteria, more than 504 experimentally verified type III secreted effector proteins have been identified that facilitate a variety of host phenotypes (http://effectors.bic.nus.edu.sg). Identification of these effector proteins and their apparent phenotypes is a major milestone; however, simply identifying the effectors has not led to new advances in disease treatment. Indeed, contact-dependent effectors that are not secreted into the extracellular space make poor targets for intervention. While determining the phenotypes attributable to specific effector proteins is useful, it becomes necessary to identify the host target proteins to gain a mechanistic understanding of effector protein function^57^.

Because the genome of *C. jejuni* does not have any apparent pathogenicity island or dedicated virulence-associated secretion system, it has been thought that *C. jejuni* was incapable of secreting and delivering effector proteins. While it is often overlooked, the flagellar apparatus is, in fact, a T3SS that functions in much the same way as a dedicated virulence-associated T3SS to secrete proteins. Studies with the *Salmonella* effector proteins SptP and SopE provide support for this observation; removing the chaperone binding domains from SptP and SpoE causes the effector proteins to be secreted through the flagellar export pathway rather than the dedicated injectisome^58^. The secretion of virulence proteins by *C. jejuni* was first described in 1999 and was found to be dependent on the flagellar secretion apparatus in 2001^21,59^. These flagellar-dependent secreted virulence proteins were termed the *Campylobacter* invasion antigens (Cia), and three of these proteins (CiaC, CiaD, and CiaI) have been demonstrated to be delivered to the cytosol of host cells^22,23^. Since the initial report of the Cia proteins, several research groups have identified additional virulence proteins secreted by the flagellar export apparatus^17,18,20^. Importantly, all of these secreted proteins mediate the interaction between *C. jejuni* and host cells.

Invasion of intestinal epithelial cells is a phenotype of several enteric pathogens that harbor effector proteins to promote cellular uptake. This strategy is an effective means to maintain residence in the intestine and avoid the innate immune response, at least temporarily^60^. Noteworthy is that the *Salmonella* effector protein SseI, which only has 11.6% identity to CiaD at the deduced amino acid level, binds to IQGAP1^61–63^. Moreover, Kim et al. (2011) reported that the ability of *Salmonella typhimurium* to invade cells and activate both Rac1 and Cdc42 was significantly attenuated in IQGAP1-null mouse embryonic fibroblasts (MEFs)^63^. However, in contrast to our study, there was a lack of Erk 1/2 activation in IQGAP1 knockout cells upon infection with *Salmonella*^63^. These findings demonstrate that while both *Salmonella* and *C. jejuni* target IQGAP1 to promote bacterial uptake, they modulate the IQGAP1-mediated signaling differently. Furthermore, our studies have uncovered the mechanistic basis for the modulation of the IQGAP1-related Rac1 signaling by *C. jejuni*, which is by displacing RacGAP1, a suppressor of Rac1 activation.

RacGAP1 modulates the activity of Rac1 during cellular migration^45,64^, which requires integrin recycling. It is thought that during integrin activation, IQGAP1 is recruited to the sites of activated integrins, activating Rac1, which further progresses into the recruitment of RacGAP1 to prevent unconstrained Rac1 activity^45,64^. Our findings support a model whereby CiaD binds to IQGAP1 and prevents, or excludes, RacGAP1 from associating with the IQGAP1 complex. This blockage of a negative regulator of Rac1 activity (a GTPase-activating protein) then promotes localized Rac1 activity. We propose that this event leads to exaggerated actin reorganization and uptake of the associated *C. jejuni*. Furthermore, normal integrin recycling would likely be disrupted, leaving activated integrins on the surface of cells. The data from our study indicate that the CiaD effector protein alters the composition and function of the IQGAP1 complex by excluding RacGAP1.

A surprising finding in our study is that *C. jejuni* activates the MAPK signaling pathway in the absence of IQGAP1. It is well established in the literature that IQGAP1 is capable of scaffolding the upstream signaling molecules MEK 1/2 as well as Erk 1/2^65^. However, because Erk 1/2 activation was independent of IQGAP1, CiaD must facilitate Erk 1/2 activation through a mechanism independent of IQGAP1. Raza and colleagues recently reported that the protein DYNLRB1, which was a high confidence hit in our Y2H screen, modulates the Erk 1/2 signaling pathway^66^. Thus, it is possible that CiaD binds to DYNLRB1 and modulates Erk 1 /2 signaling to promote *C. jejuni* uptake. Additional research may also uncover the possibility that one or more of the other proteins identified in the pull-down experiment can modulate the MAPK signaling pathway. Furthermore, while we did not directly identify a component of the MAPK signaling pathway in the pull-down or Y2H experiments performed with CiaD, it is possible that CiaD binds only transiently to a component of the MAPK pathway. Due to experimental limitations, it is likely that our experiments did not identify every binding partner of CiaD. Moreover, the diversity of proteins in the intestinal epithelium may not have been captured by the epithelial cell lysates and cDNA libraries used in this study. However, it is also clear that targeted assays have demonstrated that CiaD binds to IQGAP1, providing strong evidence for additional biological roles and binding partners for CiaD and other *C. jejuni* effector proteins.

We have demonstrated that *C. jejuni* possesses a flagellar-dependent effector that directly interacts with a host cell protein to modulate the actin cytoskeleton through the Rac1 signaling pathway. These findings are important because they demonstrate the possibility that a motile bacterium may manipulate the behavior of host cells through flagellar-secreted effectors. There is remarkable progress being made using information from highly studied bacteria that have dedicated T3SSs. However, there is nearly no information about secreted effectors in bacteria lacking dedicated secretion systems. Moreover, secreted bacterial effector proteins are still nearly impossible to identify through sequence analysis alone. In this study, we have identified a host cell target of a *C. jejuni* flagellar-secreted protein. The identification of IQGAP1 is particularly interesting because it is a well-known regulator of signaling events involved in cytoskeletal rearrangement. Moreover, this finding, in combination with the results from inhibitor studies and knockdown experiments that demonstrate the necessity of focal adhesion components, provides a framework for molecular dissection of *C. jejuni* invasion. Our findings also raise the possibility that numerous pathogenic and commensal bacteria alike may have secreted effector proteins hidden in their genomes that are modifying the behavior of the host cells.

## Methods

### Bacterial culture conditions

The *C. jejuni* F38011^67^ and 81–176^68^ strains were cultured on Mueller-Hinton agar plates supplemented with 5% citrated bovine blood (MHB) at 37 °C in a microaerobic environment (5% O_2_, 10% CO_2_, 85% N_2_). The *C. jejuni* F38011 *ciaD* deletion mutant and complemented isolate was generated as described previously^23^. The *C. jejuni* 81–176 ∆*ciaD* strain was created using the same plasmid described previously^23^, with the tetracycline resistance cassette swapped for a chloramphenicol resistance cassette at the SacII sites. Where necessary, tetracycline (128 µg/mL) or chloramphenicol (8 µg/mL) was added to the growth media. All *Escherichia coli* strains were grown on LB agar plates at 37 °C supplemented with carbenicillin (100 µg/mL) as necessary.

### Cell culture

Human INT 407 cells (ATCC CCL-6) were routinely cultured at 37 °C in a 5% CO_2_ incubator in Eagles MEM (Gibco, Waltham, MA) supplemented with 10% FBS (Cat# 97068-091, VWR, Radnor, PA).

### CiaD pull-down assays and sample preparation

CiaD was cloned into the EcoRI and XhoI sites of pGEX-5X-1 with the primers 5′-ATA ATG AAT TCA TGA ATT TGG AAG ATT TAG CTA AAA AAA C-3′ and 5′-ATA ATC TCG AGA AGC TTA TCT TCG ATA TTT GCA AGC-3′ (Also indicated in Supplementary Table 1). The CiaD-GST fusion was expressed in *E. coli* BL21(DE3) and induced with 1 mM of Isopropyl-β-D-thiogalactoside (IPTG) for 16 h at 4 °C. Host-interacting proteins were collected by loading CiaD onto a glutathione-S-transferase (GST) column and adding an INT 407 cell lysate. Proteins were eluted using reduced glutathione. Negative controls were performed by loading GST alone, expressed from pGEX-5X-1. A Pierce^TM^ BCA Protein Assay Kit (Thermo Scientific, Waltham, MA) was used to determine the concentration of the proteins in the pull-down eluate. The eluates were denatured for 30 min at 58 °C in the following solution: 500 mM of ammonium bicarbonate, 8 M urea, and 500 mM of DTT. The sulfur groups of cysteine were then derivatized using 400 mM Iodoacetamide for 30 min at 37 °C. Trypsin was added to the denatured proteins at a sample protein to trypsin ratio of 50:1. The proteins were digested for 4 h at 37 °C and were desalted on a reverse-phase Supelco C18 column and stored at −80 °C before analysis.

### LC–MS/MS analysis of the pull-down samples

The liquid chromatography (LC) system was custom-built, comprising of a PAL autosampler (Leap Technologies, Carrboro, NC), two Agilent 1200 nanoflow pumps and one Agilent 1200 capillary pump (Agilent Technologies, Santa Clara, CA), and multiple Valco valves (Valco Instruments Co., Houston, TX). Reversed-phase columns were prepared in-house by slurry packing 3 µm Jupiter C_18_ (Phenomenex, Torrance, CA) into 40 cm × 360 µm outer diameter (OD) × 75 µm inner diameter (ID) fused silica (Polymicro Technologies Inc., Phoenix, AZ) using a 1 cm sol-gel frit for media retention^69^. Trapping columns were prepared similarly by slurry packing 5 µm Jupiter C_18_ into a 4 cm length of 150 µm ID fused silica and fritted on both ends. 5 µL of a 0.1 µg/µL peptide solution was injected for each sample prepared. The samples were trapped and washed on the columns at 3 µL/min for 20 min prior to alignment with analytical columns. Mobile phases consisted of 0.1% formic acid in water (A) and 0.1% formic acid acetonitrile (B) operated at 300 nL/min with a gradient profile as follows (min:%B); 0:5, 2:8, 20:12, 75:35, 97:60, 100:85.

MS analysis was performed using a Velos Orbitrap mass spectrometer (Thermo Scientific, San Jose, CA) outfitted with a custom electrospray ionization (ESI) interface. Electrospray emitters were custom made by chemically etching 150 µm OD × 20 µm ID fused silica^70^. The heated capillary temperature and spray voltage were 350 °C and 2.2 kV, respectively. Data were acquired for 100 min after a 15 min delay from the start of the gradient. Orbitrap spectra (AGC 1 × 106) were collected from 400–2000 m/z at a resolution of 60k followed by data-dependent ion trap MS/MS (collision energy 35%, AGC 1 × 104) of the ten most abundant ions. A dynamic exclusion time of 60 s was used.

### Proteomics data analysis

The ‘Thermo.Raw’ files were analyzed with MS-GF+^71^ against a database comprising the Uniprot non-redundant SwissProt human proteome (20,216 sequences, downloaded in April 2015) and the sequence of *Campylobacter* CiaD. MS-GF+ parameters were the following: semi-tryptic peptides were authorized, methionine oxidation was set as variable modification, cysteine carbamidomethylation was set as fixed modification. A decoy database was used to generate false-positive identifications. Results were filtered to have a false discovery rate below 1%. Using a MS-GF+ score below 4.77E-10 resulted in a False Discovery Rate below 1% at the spectral, peptide and protein level (proteins were only considered identified when at least 2 unique peptides were attributed to them). A spectral counting strategy was used to estimate abundance all the unique peptides attributed to a given protein were used for quantification (e.g., unmodified, modified, tryptic and semi-tryptic peptides). Only proteins with at least 10 spectral counts in any of the three pull-down experiments were considered for quantification.

### Yeast two-hybrid screening

Yeast two-hybrid screening was performed by Hybrigenics Services, S.A.S., Evry, France (http://www.hybrigenics-services.com). *C. jejuni ciaD* gene was PCR-amplified and cloned into the plasmid pB27 as a C-terminal fusion to LexA (LexA-CiaD). The construct was checked by sequencing the entire insert and used as a bait to screen a random-primed placenta cDNA library constructed into the plasmid pP6. pB27 and pP6 derive from the original pBTM116^72^ and pGADGH^73^ plasmids, respectively. A total of 104 million clones (10-fold the complexity of the library) were screened using a mating approach with YHGX13 (Y187 ade2-101::loxP-kanMX-loxP, matα) and L40ΔGal4 (mata) yeast strains as described previously^74^. 285 His+ colonies were selected on a medium lacking tryptophan, leucine, and histidine, and supplemented with 2 mM 3-aminotriazole to handle bait autoactivation. The prey fragments of the positive clones were amplified by PCR and sequenced at their 5′ and 3′ junctions. The resulting sequences were used to identify the corresponding interacting proteins in the GenBank database (NCBI) using a fully automated procedure. A confidence score (Predicted Biological Score) was attributed to each interaction, as previously described^75^.

The Predicted Biological Score relies on two different levels of analysis. Firstly, a local score takes into account the redundancy and independency of prey fragments, as well as the distribution of reading frames and stop codons in overlapping fragments. Secondly, a global score takes into account the interactions found in all the screens performed at Hybrigenics using the same library. This global score represents the probability of an interaction being nonspecific. For practical use, the scores were divided into four categories, from A (highest confidence) to D (lowest confidence). A fifth category (E) specifically flags interactions involving highly connected prey domains previously found several times in screens performed on libraries derived from the same organism. Finally, several of these highly connected domains have been confirmed as false-positives of the technique and are now tagged as F. The Predicted Biological Score scores have been shown to positively correlate with the biological significance of interactions^76,77^.

### Purification of CiaD, CiaC and FlpA recombinant proteins for pull-down assays

*C. jejuni* CiaD, CiaC and FlpA recombinant proteins were purified by using the following expression vectors; pMCGS68 for His6-CiaD and pET-24b(+) for His6-CiaC and His6-FlpA. Briefly, the *E. coli* BL21 (DE3) cells containing either plasmid pMCGS68-His6-CiaD, pET24b-His6-CiaC, or pET24b-His6-FlpA were grown in LB broth for overnight at 37 °C supplemented by either ampicillin (for pMCGS68-His6-CiaD) or kanamycin (for pET-24b-His6-CiaC and pET-24b-His6-FlpA). The overnight cultures were inoculated in two 250 mL LB broth at a ratio of 1:50 and incubated at either 25 °C (for pMCGS115-His6-CiaD) or at 37 °C (for pET-24b-His6-CiaC and pET-24b-His6-FlpA) with appropriate antibiotic selection. After 4 h of incubation or when the culture was in the exponential growth phase (OD_600_ = 0.35–0.5), 1 mM IPTG was added into the culture for induction and incubated for another 4 h at the appropriate condition. After the induction, cells were pelleted by centrifugation and mixed with IP buffer (25 mM Tris, 150 mM NaCl, 1% Triton X-100, 5% Glycerol, 1 mM PMSF) and lysed by sonication with appropriate settings. The cell lysates were cleared by centrifugation followed by filtration through a 0.45 µm syringe filter. The clear cell lysates were passed through the 2 mL TALON metal affinity resins (Takara Bio) in a 10 mL chromatography column (Bio-Rad). The eluted products were further cleared and concentrated by using the Amicon Ultra 10,000 MW filter (EMD Millipore). A BCA assay was done to determine the protein concentration. The purity of the protein products were determined by sodium dodecyl sulfate-polyacrylamide gel electrophoresis (SDS-PAGE).

### Pull-downs with purified CiaD, CiaC and FlpA

Pull-down assays were performed on a 10 mL chromatography column (Bio-Rad). Briefly, 100 µL of TALON metal affinity resins were added to the column followed by washing the beads with 10 mL wash buffer (25 mM Tris, 150 mM NaCl). An equal amount of purified recombinant His6-CiaD, His6-CiaC, and His6-FlpA proteins or bovine serum albumin (BSA, negative control) were suspended in 5 mL IP wash buffer (25 mM Tris, 150 mM NaCl, 1% Triton X-100, 5% Glycerol) and added to a column. The flow-through of the proteins were loaded into the columns two more times for optimal binding of His-tagged proteins to the TALON resin. Then, equal amount of INT 407 cell lysates at a ratio of 1: 4 of the purified protein was loaded onto the column. The flow-through of the lysates was loaded four more times for optimal binding of lysates to either CiaD, CiaC, FlpA, or BSA. Finally, the columns were washed with 10 mL IP wash buffer (25 mM Tris, 300 mM NaCl, 0.1% Triton X-100, 5% Glycerol) followed by elution with 5 mL elution buffer (150 mM imidazole, 25 mM Tris, 300 mM NaCl, 0.1% Triton X-100, 5% Glycerol). The eluted proteins were concentrated by using the Amicon Ultra-filter by centrifugation at 3000 × *g* for 40 min. Samples were suspended in 2X sample buffer and boiled prior to immunoblot analysis. The immunoblots were probed with mouse anti-IQGAP1 antibody (sc-376021, Santa Cruz Biotechnology) at a dilution of 1:1000. An anti-mouse IgG (A4416, Sigma) was used for the secondary antibody at a dilution of 1:4000 and the ECL Prime Detection Reagent (Amersham) was used to visualize the immunoblot.

### Purification of IQGAP1-GRD

The gene sequence encoding IQGAP1-GRD was amplified from the pCMV-IQGAP1-Myc plasmid (see below) using the forward primer: 5′-GGA TCC GAA TTC GAG CTC CGA GAA GAG AGA GAA GTT GGA AGC TTA CC-3′ and the reverse primer: 5′-GTG CTC GAG CTT GTC ATC GTC ATC CTT GTA GTC GTC GAC CAG GGT GAG AGA CAC TTC CGT-3′, resulting in a FLAG-tagged gene fragment. This fragment was cloned into the pET24b vector for expression in *E. coli*. Protein was produced by adding 1 mM IPTG to log-phase *E. coli* and incubating at 37 °C for 4 h. *E. coli* was lysed in IP Wash Buffer, and protein was collected by running the lysate over a TALON affinity Column. Proteins were eluted in elution buffer for further use.

### IQPAG1-GRD interaction ELISA

Polystyrene plates were coated with 3 µg per well of either His6-CiaD, His6-CiaC, His6-FlpA, or BSA (negative control) overnight in 0.1 M carbonate buffer (pH 9.5) at 4 °C. Plates were washed in PBS + 0.1% Tween buffer (above), followed by blocking with 1% BSA (w/v) in PBS. IQGAP1-GRD was added to the wells in 2-fold serial dilutions from 25 µg/mL to 0.390625 µg/mL and incubated for 2 h at room temperature. Wells were then washed in PBS + 0.1% Tween buffer before adding anti-FLAG antibody (Cat #F3165, Sigma-Aldrich) at a concentration of 1:2000, followed by a two-hour incubation at room temperature. Wells were washed, and secondary HRP-conjugated anti-mouse antibody was added (Cat # A4416, Sigma-Aldrich) at a concentration of 1:4000. After a two-hour incubation, wells were washed with PBS + 0.1% Tween and developed with TMB substrate according to the manufacturer’s instructions (Cat # 34021, Thermo Fisher). Wells were read in a BioTek EL_X_808 plate reader at 450 nm.

### INT 407 lysate preparation

INT 407 cells were harvested with trypsin, pelleted by centrifugation, and washed with 10 mL of PBS containing EDTA-free protease inhibitor cocktail (Halt™ Protease Inhibitor Cocktail, EDTA-Free, Thermo Fisher Scientific, Waltham, MA). The cell pellet was then resuspended in 1–3 mL of immunoprecipitation (IP) buffer (25 mM Tris pH 7.5, 150 mM NaCl, 5% Glycerol, 1% Triton X-100) with protease inhibitor cocktail and lysed by repeated passage through a 21-gauge needle. Whole-cell lysates (WCLs) were clarified by centrifugation at 14,000 × *g* for 20 min at 4 °C. Total protein content was determined by a BCA assay.

### Pull-downs with purified CiaD

Purified recombinant His6-CiaD protein or an equivalent volume of PBS (negative control) was added to an INT 407 lysate at a ratio of 4 times more His6-CiaD protein than INT 407 protein. The mixed sample was incubated with rotation at 4 °C for 4 h. Following incubation, 50 µL of IP buffer-equilibrated TALON metal affinity resin was added and incubated for 4 h with rotation at 4 °C. After incubation, the TALON resin was collected by centrifugation at 2,000 × *g* for 5 min, and proteins were suspended in 2X sample buffer and boiled prior to immunoblot analysis.

### Creation of stable cell lines

In a 6-well tissue culture dish, an overnight culture of INT 407 cells (4 ×105 cells per well) was infected with approximately 10^6^ Lentiviral transduction particles (Mission, Sigma) in MEM supplemented with 10 µg/mL polybrene (Sigma, St. Louis, MO). Cells were infected with lentiviral particles carrying shRNA that was non-targeting (Sigma Cat# SHC016V) or targeting IQGAP1 (Sigma Mission shRNA library cat # TRCN0000298928, TRCN0000298930). Throughout the paper, these are referred to as ‘928’ and ‘930’. Cells were incubated for two days with the virus, and the medium was changed for MEM supplemented with 2.5 µg/mL puromycin (Sigma). After two days of selection, cells were trypsinized and seeded at ~1 cell/well in a 96-well tissue culture plate in MEM with puromycin. Only wells that were verified by microscopy to contain one INT 407 cell were used for clonal expansion. The expression of IQGAP1 in the clonal cell lines was determined by SDS-PAGE and immunoblot analysis, as described below.

### IQGAP1 plasmid construction

The IQGAP1 cDNA was amplified from the plasmid HsCD00347159 from the Harvard PlasmID database using the primers 5′- GCC ATG GAG GCC CGA ATT CGG ATG TCC GCC GCA GAC GAG GTT -3′ and 5′- GAT CCC CGC GGC CGC GGT ACC TTA CTT CCC GTA GAA CTT TTT GTT GAG AA -3′. In-Fusion cloning was used to insert IQGAP1 into the pCMV-Myc vector that was linearized using EcoRI and KpnI. The pCMV-Myc-EGFP plasmid was generated by insertion of the EGFP gene into the SalI and NotI restriction sites of the pCMV-Myc vector.

### Gentamicin-protection assays

The gentamicin protection assay was used to assess cellular adherence and internalization in knockdown cell lines, as described previously^78^. Briefly, 1.5 × 10^5^ of each cell line was seeded in eight wells of a 24-well tissue culture tray overnight. Cells were rinsed with MEM containing 1% FBS and infected with ~1 × 10^7^ CFU of *C. jejuni* (OD_540_ = 0.03). The adherence assays were performed by incubation of *C. jejuni* with the epithelial cells for 3 h. The cells were then rinsed three times with PBS, lysed with 0.1% Triton X-100, and samples serially diluted and spread onto MHB plates. The invasion assays were performed by incubating *C. jejuni* with the cells for 3 h, washing the cells three times with PBS, and adding MEM with 250 µg/mL of gentamicin for an additional 3 h to kill extracellular bacteria. Following incubation, the cells were rinsed three times with PBS, lysed with 0.1% Triton X-100, and samples were serially diluted and spread onto MHB plates. The numbers of adherent (cell-associated) and internalized bacteria were determined by counting the resulting colonies. To test if the expression of IQGAP1 rescued invasion in the knockdown cell lines, cells were seeded at 0.7 × 10^5^ cells/well in a 24-well tissue culture tray and transfected with pCMV-IQGAP1-Myc or pCMV-EGFP-Myc the following day using Lipofectamine 3000 (Thermo Fisher Scientific) following the manufacture’s instructions. Similarly, the effects of Rac1 activation and Cdc42 activation were tested by transfecting the cells with plasmids expressing GFP tagged wild-type Rac1, constitutive-active Rac1 (L61), dominant-negative Rac1 (N17), wild-type Cdc42, constitutive-active Cdc42 (L61), and dominant-negative Cdc42 (N17)^79^. Approximately 18 h after transfection, the adherence and internalization assays were performed as outlined above. All assays were performed in biological triplicate, and representative assays are shown. Cell viability was determined by collecting three triplicate wells of transfected cells by trypsinization, staining with trypan blue, and counting the cells in an EVE Automated Cell Counter (NanoEnTek, Inc, Waltham, MA).

### Confocal microscopy examination of *C. jejuni* infected IQGAP1 transfected cells

INT 407 IQGAP1 knockdown cells (930-C5) were seeded at a density of 0.5 × 10^5^ cells on 10 mm round coverslips in MEM with 10% FBS. After overnight incubation, the cells were transfected with pCMV-IQGAP1-Myc using Lipofectamine 3000 (Thermo Fisher Scientific). Approximately 18 h after transfection, cells were washed in MEM with 1% FBS and infected with ~1 × 10^5^ CFU of *C. jejuni* (OD_540_ = 0.003) for 10 h. The cells were fixed with 4% paraformaldehyde in PBS for 5 min, permeabilized using PBS with 0.1% Triton X-100 and 0.3% BSA, and then stained with antibodies. Primary antibodies against *C. jejuni* (1:4000, rabbit polyclonal)^80^ and c-Myc (1:500 mouse monoclonal, Takara Bio) were added for 45 min. After washing three times with PBS, AlexaFlour 680 anti-rabbit and AlexaFlour 488 anti-mouse secondary antibodies (1:1000, Jackson ImmunoResearch, West Grove, PA), along with Texas Red-X conjugated phalloidin (1:1000, Thermo Fisher Scientific), were added for 30 min. Coverslips were washed three times with PBS and mounted on slides using ProLong Diamond Antifade Mountant (Thermo Fisher Scientific). After curing overnight, slides were imaged using a Leica SP5 laser scanning confocal microscope. Images were quantitated by counting *C. jejuni* in ImageJ.

### Erk 1/2, Cdc42, and Rac1 activation experiments

Stable human INT 407 cells expressing a non-targeting shRNA or an shRNA targeting IQGAP1 were seeded to a density of 1 × 10^5^ cells per well in a 24-well tissue culture tray in MEM supplemented with 10% FBS. Prior to *C. jejuni* infection, the cells were serum-starved in MEM without FBS for 4 h. An overnight biphasic culture of *C. jejuni* was then used to infect the cells at an OD_540_ of 0.3. For testing of Erk 1/2 activation, samples were collected, and immunoblots were performed as described below. For testing of Cdc42 and Rac1 activation, samples were collected and processed in accordance with the instructions provided with the Cdc42 G-LISA and Rac1 G-LISA kits (Cytoskeleton Inc., Denver, CO). The cell lysates were adjusted to a concentration of 0.4 mg/mL prior to the assays. Samples were flash-frozen in liquid nitrogen and stored at −80 °C. Three biological samples were collected on different days for analysis.

### Immunoprecipitating the IQGAP1 complex

INT 407 cells were seeded at a density of 5.5 × 10^6^ cells in a 100 mm dish and incubated overnight. The cells were then serum-starved for three hours, followed by infection with *C. jejuni* at an OD_540_ of 0.35 in 10 mL of MEM 1% FBS. After 45 min of infection, cells were lysed on ice in IP buffer (see above) with 50 mM NaCl that was supplemented with a protease inhibitor cocktail and phosphatase inhibitor cocktail (Halt Phosphatase Inhibitor Cocktail, Pierce, Rockford IL). Lysates were clarified by centrifugation at 13,000 × *g*. IQGAP1 complexes were immunoprecipitated by incubating the clarified lysates with mouse anti-IQGAP1 (Clone C-9, Santa Cruz Biotechnology, Dallas, TX) overnight at 4 °C with rotation, followed by overnight incubation with Protein A/G agarose (Santa Cruz Biotechnology). Beads were collected by centrifugation at 2,000 × *g* and washed two times in IP buffer with 50 mM NaCl. Proteins were dissociated from the beads by boiling in 2X sample buffer for five minutes before analysis by immunoblot (as described below). Antibodies were used to detect RacGAP1 (Thermo Fisher), IQGAP1 (Abcam, Cambridge, MA), Rac1 (Cell Signaling Technology), and Erk 1/2 (Santa Cruz Biotechnology).

### Immunoprecipitating IQGAP1 using *C. jejuni* produced CiaD

For IQGAP1 co-immunoprecipitation assays with *C. jejuni* expressing CiaD, a 60% confluent 100-mm dish of INT 407 cells were infected with a *C. jejuni ciaD* deletion mutant harboring a plasmid containing either *ciaD-*FLAG or *ciaD∆*MKD-FLAG. The construction of these recombinant strains was described previously^23^. The cells were infected in MEM with 1% FBS at an OD_540_ of 0.3 for 2 h before lysis in IP buffer (see above) on ice. The entire contents of the dish were harvested by scraping in 1 mL of cold IP buffer and then centrifuged at 13,000 × *g* to clarify the lysate. The lysate was incubated with anti-FLAG M2 affinity gel (Sigma) at 4 °C overnight. Samples were washed three times in IP buffer, placed in a boiling water-bath for 5 min and subjected to discontinuous SDS-PAGE in a 10% polyacrylamide gel. The proteins were transferred to membranes and probed with IQGAP1 and FLAG antibodies.

### Immunoblotting and SDS-PAGE

For immunoblotting IQGAP1, phospho-Erk 1/2, and total-Erk 1/2, cell lysates were collected from 24-well plates by adding 300 µL of single-strength sample buffer and agitation. Samples were boiled for 5 min, and proteins were separated by SDS-PAGE with 12.5% polyacrylamide described by Laemmli^81^. Gels were either stained with Coomassie brilliant blue R250 (CBB-R250) or transferred to polyvinylidene fluoride (PVDF) membranes. Membranes were blocked with 5% non-fat dry milk and incubated with antibodies against IQGAP1 (1:1000, Abcam) or total-Erk 1/2 (1:2000 Santa Cruz Biotechnology). Membranes probed with antibodies against phospho-Erk 1/2 (1:2000 Cell Signaling Technology) with blocked with 5% BSA. The blots were then incubated with a goat anti-rabbit antibody conjugated to horseradish peroxidase (1:2500, Sigma), and reactive bands were visualized using Amersham ECL prime reagent (GE Healthcare). Images of blots were acquired on a GE Healthcare ImageQuant LAS 4000 Mini system.

### Statistics and reproducibility

All statistical analysis presented was performed with GraphPad Prism 6.0 g (La Jolla, CA). Unless indicated otherwise, all experiments presented were performed at in at least three independent replicates.

### Reporting summary

Further information on research design is available in the Nature Research Reporting Summary linked to this article.

## Supplementary information

Supplementary Information

Description of Additional Supplementary Files

Supplementary Data 1

Supplementary Data 2

Reporting Summary

## Data Availability

Mass spectrometric raw data were deposited in the MassIVE (https://massive.ucsd.edu/) repository, a member of the proteomeXchange consortium, under the accession number MSV000086662. The identified peptide, protein, and quantified protein lists with expression values and associated statistics are attached to this manuscript in the Supplementary Data 1. Any plasmids, mutants, or cell lines created for this study will be made available upon request. Source data are provided with this paper.

## Source data

Source Data
