# Supplementary material for: The Campylobacter jejuni CiaD effector co-opts the host cell protein IQGAP1 to promote cell entry
Source: Nat Commun. 2021 Feb 26;12:1339. doi: 10.1038/s41467-021-21579-5 (PMC7910587; doi:10.1038/s41467-021-21579-5)

Supplementary Information

The *Campylobacter jejuni* CiaD effector co-opts the host cell protein IQGAP1 to promote cell entry

Nicholas M. Negretti[1], Christopher R. Gourley[1], Prabhat K. Talukdar[1], Geremy Clair[2], Courtney M. Klappenbach[1], Cody J. Lauritsen[1], Joshua N. Adkins[2], Michael E. Konkel[1*]

[1] School of Molecular Biosciences, College of Veterinary Medicine, Washington State University, Pullman, WA, United States, [2] Integrative Omics, Pacific Northwest National Laboratory, Richland, WA, United States

Supplementary Table 1. Primers.

Name	Sequence
pGEX-5X-1-CiaD-F	5'- ATA ATG AAT TCA TGA ATT TGG AAG ATT TAG CTA AAA AAA C -3'
pGEX-5X-1-CiaD-R	5'- ATA ATC TCG AGA AGC TTA TCT TCG ATA TTT GCA AGC -3'
pET24b-IQGAP1-GRD-F	5’-GGA TCC GAA TTC GAG CTC CGA GAA GAG AGA GAA GTT GGA AGC TTA CC-3’
pET24b-IQGAP1-GRD-R	5’-GTG CTC GAG CTT GTC ATC GTC ATC CTT GTA GTC GTC GAC CAG GGT GAG AGA CAC TTC CGT-3’
pCMV-Myc-IQGAP1-F	5'- GCC ATG GAG GCC CGA ATT CGG ATG TCC GCC GCA GAC GAG GTT -3'
pCMV-Myc-IQGAP1-R	5'- GAT CCC CGC GGC CGC GGT ACC TTA CTT CCC GTA GAA CTT TTT GTT GAG AA -3'

Supplementary Figure 1. IQGAP1 contains multiple functional domains. Panels: **(A)** IQGAP1 comprises a calponin homology domain (CH), a coiled-coil domain (CC), a tryptophan containing domain (WW), four isoleucine/glutamine containing domains (IQ), a Ras-GAP domain (GRD), and a Ras-GAP C terminus domain (RGCT). The amino acid start and stop positions of all domains are listed, and it was found that the CiaD protein interacted with a fragment of IQGAP1 containing the amino acids 964 through 1365. **(B)** CiaD binding regions identified by the yeast-two hybrid screen do not have similar protein sequences. The CiaD interacting regions of IQGAP1 (amino acids 964 to 1365), NRBP1 (amino acids 457 to 523), and DYNLRB1 (entire protein) were aligned using M-Coffee. Protein-protein alignments did not reveal any regions of significant similarity between the interacting regions of the three proteins, indicating that either CiaD is capable of binding to multiple different amino acid motifs or that interactions between CiaD and host proteins are mediated by tertiary protein structure rather than sequence alone.

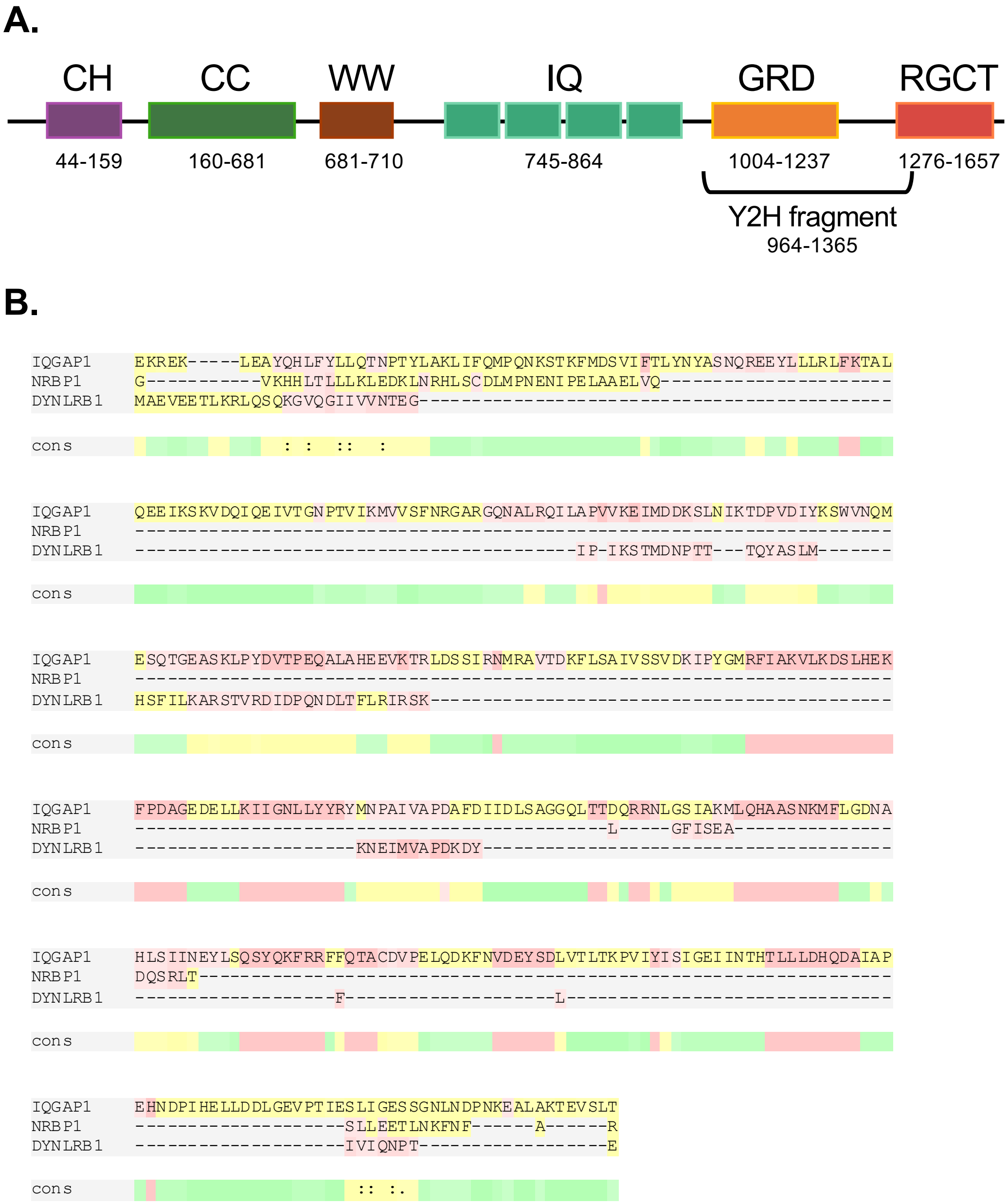

Supplementary Figure 2. IQGAP1 is specifically pulled down with CiaD and not other *C. jejuni* proteins. Recombinant His6-CiaD, His6-CiaC, and His6-FlpA was purified from *E. coli* and then mixed with a whole-cell lysate (WCL) prepared from INT 407 cells. The recombinant His6 tagged proteins were then pulled down using Talon affinity resin. Panels: **(A)** The protein mixtures were separated by SDS-PAGE and stained with Coomassie Brilliant Blue R-250, where the recombinant proteins are visible. **(B)** The samples were immunoblotted with anti-IQGAP1 antibodies. IQGAP1 was pulled down in the presence of CiaD but not in the presence of CiaC, FlpA, or in the absence of proteins (BSA + WCL). IQGAP1 is present in the INT 407 input lysate (WCL) used for the pull-down input. Molecular weight in kDa is indicated.

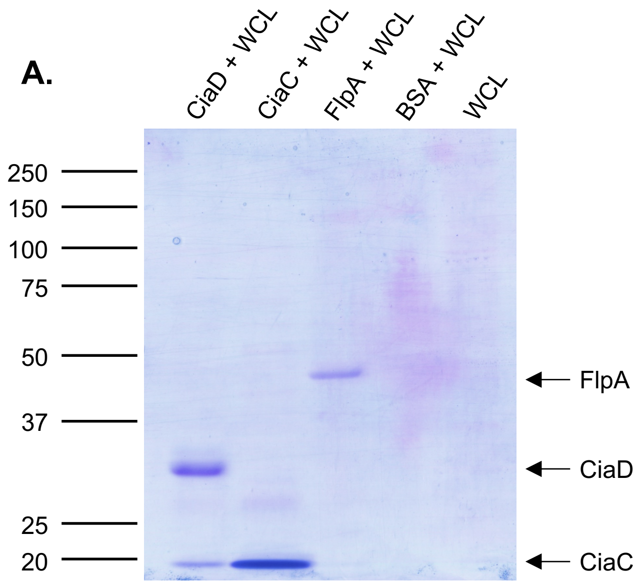

Supplementary Figure 3. IQGAP1 is required for cellular invasion by *C. jejuni.* Stable cell lines expressing two different shRNA constructs that target IQGAP1 were generated. The shRNA labeled ‘928’ targets the coding sequence of IQGAP1, while ‘930’ targets the 3’-UTR. Labels after the number indicate the individual clone. The ability of *C. jejuni* to adhere and invade the cell lines was tested using the gentamicin protection assay. Panels: **(A)** Knockdown of IQGAP1 does not alter the cellular adherence of *C. jejuni* (n = 3 independent samples). Error bars represent standard deviation. **(B)** Knockdown of IQGAP1 reduces *C. jejuni* internalization. Error bars represent standard deviation. Compared to cells expressing non-targetting shRNA, there was reduction in invasion in 928-G10 (p = 0.0173), 928-C9 (p = 0.0678), 930-C5 (p = 0.0008), 930-D11 (p = 0.0007). **(C)** Immunoblots were performed with anti IQGAP1 antibodies on the individual cell lines to ensure that protein production was reduced (n = 3 independent samples). **(D)** Actin was used as a loading control for the cell lysate. Differences were compared against the non-targeting shRNA and the knockdown cells using an ANOVA on log-transformed counts followed by a two-tailed Sidak's test (* $p < 0.05$). For panels C and D, molecular weights are indicated in kDa.

A.

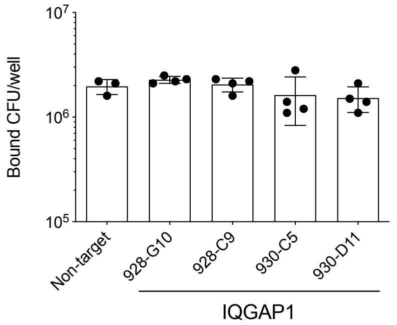

B.

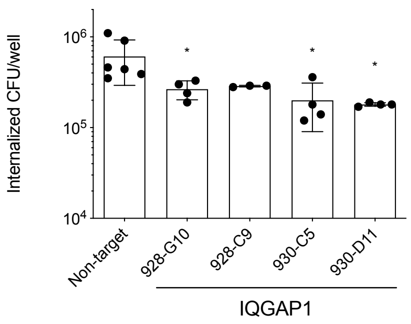

C.

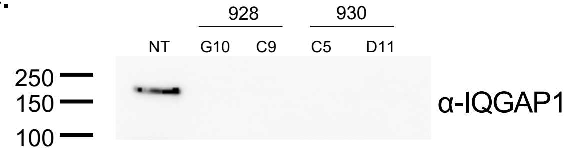

D.

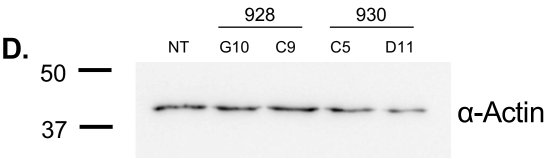

Supplementary Figure 4. Transfected cells were tested for toxicity and transfection efficiency. Panels: **(A)** There are no significant differences in cell viability among the wells transfected with different wild-type (WT), dominant negative (DN), and constitutively active (CA) constructs of Cdc42 and Rac1. Cells were trypsinized from three replicate wells and stained with trypan blue viability stain. Differences among groups were tested by ANOVA (p = 0.18). **(B)** The transfection efficiency was measured by imaging the cells and counting the number of GFP positive cells and dividing this by the total cell number in a field of view. The transfection efficiency was not found to be significantly different for any condition as tested by ANOVA (one-sided F distribution) (p = 0.1430).

A.

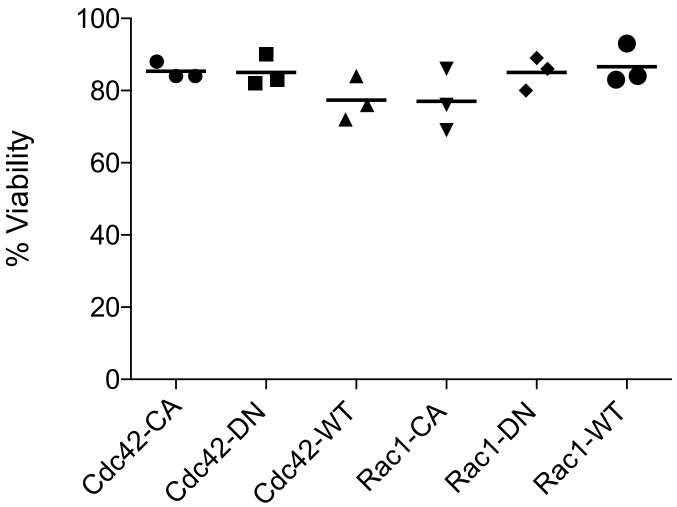

B.

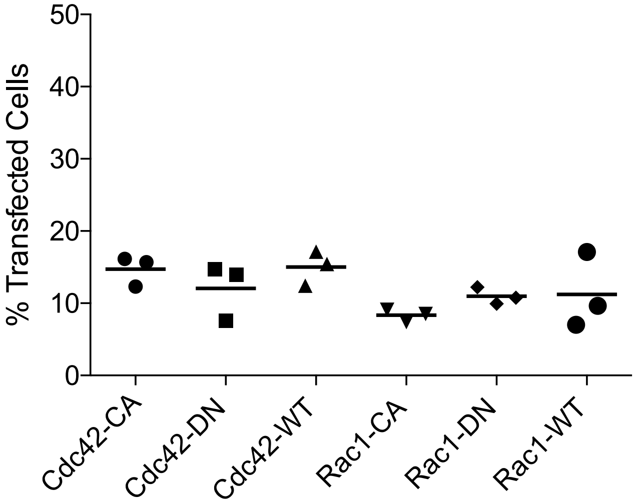

Supplement: Supplementary file 1 — Supplementary Information [file 41467_2021_21579_MOESM1_ESM.pdf]
