# Supplementary material for: The Campylobacter jejuni CiaD effector co-opts the host cell protein IQGAP1 to promote cell entry
Source: Nat Commun. 2021 Feb 26;12:1339. doi: 10.1038/s41467-021-21579-5 (PMC7910587; doi:10.1038/s41467-021-21579-5)
Supplement: Supplementary file 2 — Description of Additional Supplementary Files [file 41467_2021_21579_MOESM2_ESM.pdf]

Description of Additional Supplementary Files

File Name: Supplementary Data 1
Description: Results from the LC-MS/MS analysis of protein pull-down experiments, and is the raw data for Figure 1.

File Name: Supplementary Data 2
Description: Results from the yeast two hybrid screening of CiaD against a human protein library.
