# Supplementary material for: The Campylobacter jejuni CiaD effector co-opts the host cell protein IQGAP1 to promote cell entry
Source: Nat Commun. 2021 Feb 26;12:1339. doi: 10.1038/s41467-021-21579-5 (PMC7910587; doi:10.1038/s41467-021-21579-5)
Supplement: Supplementary file 4 — Supplementary Data 2 [file 41467_2021_21579_MOESM4_ESM.pdf]

Results Summary

ULTImate Y2H SCREEN
Campylobacter jejuni - CiaD
# vs Human Placenta_RP6

Fri, Jun 30, 2017 - 05:31 PM

Screen Parameters

Nature	cDNA
Reference Bait Fragment	Campylobacter jejuni - CiaD (aa 1-163) ; hgx4788v1
Prey Library	Human Placenta_RP6
Vector(s)	pB27 (N-LexA-bait-C fusion)
Processed Clones	285 (pB27_A)
Analyzed Interactions	104 millions (pB27_A)
3AT Concentration	2.0 mM (pB27_A)

Global PBS®

Global PBS (for Interactions represented in the Screen)		Nb	%
A	Very high confidence in the interaction	3	15.0%
B	High confidence in the interaction	1	5.0%
C	Good confidence in the interaction	0	0.0%
D	Moderate confidence in the interaction<br>This category is the most difficult to interpret because it mixes two classes of interactions :<br>- False-positive interactions<br>- Interactions hardly detectable by the Y2H technique (low representation of the mRNA in the library, prey folding, prey toxicity in yeast)	9	45.0%
E	Interactions involving highly connected (or relatively highly connected) prey domains, warning of non-specific interaction. The total number of screens performed on each organism is taken into account to set this connectivity threshold: 20 interactions to different bait proteins in our entire database for Human, 10 for Mouse, Drosophila and Arabidopsis and 6 for all other organisms. They can be classified in different categories:<br>- Prey proteins that are known to be highly connected due to their biological function<br>- Proteins with a prey interacting domain that contains a known protein interaction motif or a biochemically promiscuous motif	6	30.0%
F	Experimentally proven technical artifacts	1	5.0%
Non Appliable			
N/A	The PBS is a score that is automatically computed through algorithms and cannot be attributed for the following reasons :<br>- All the fragments of the same reference CDS are antisense<br>- The 5p sequence is missing<br>- All the fragments of the same reference CDS are either all OOF1 or all OOF2<br>- All the fragments of the same reference CDS lie in the 5' or 3' UTR		

HYBRIGENICS CONFIDENTIAL - 1104538 - M. Konkel

Prey Fragment Analysis

Symbols	Means
	The fragment contains the full length CDS
	Fragment is fully in 5' UTR
	Fragment is fully in 3' UTR
	Fragment contains at least one In Frame STOP codon
[NR]	Fragment was found to be non relevant (poor quality, high N density)
IF<br>OOF1<br>OOF2	With regard to the theoretical frame of each corresponding CDS (GeneBank),fragments are cloned in frame (IF) if they are in the same frame as Gal4AD.In general, polypeptides synthesized from OOF fragments are not considered of biological interest, unless found together with another frame. However, some of the proteins expressed from an OOF fragment can be translated in the correct frame,due to the existence of natural frame-shift events during translation in yeast
??	Unidentified frame when :<br>- The clone sequence is antisense<br>- The 5p sequence is missing
N	Antisense
Start...Stop	Position of the 5p and 3p prey fragment ends, relative to the position of the ATG start codon (A=0)

Clone Name	Type Seq	Gene Name (Best Match)	Start..Stop (nt)	Frame	Sense	%Id 5p	%Id 3p	PBS
pB27_A-105	5p/3p	Homo sapiens - BRAF	322..982	OOF1		94.5	94.3	N/A
pB27_A-75	5p/3p	Homo sapiens - DHX9	1026..2363	IF		99.7	100.0	D
pB27_A-277	5p/3p	Homo sapiens - DHX9	1026..2363	IF		100.0	100.0	D
pB27_A-27	5p/3p	Homo sapiens - DHX9	1026..2363	IF		99.9	98.3	D
pB27_A-42	5p/3p	Homo sapiens - DHX9	1026..2363	IF		99.9	99.8	D
pB27_A-294	5p/3p	Homo sapiens - DYNLRB1	-208..234	IF		100.0	100.0	A
pB27_A-36	5p/3p	Homo sapiens - DYNLRB1	-208..234	IF		100.0	99.8	A
pB27_A-2	5p/3p	Homo sapiens - DYNLRB1	-208..234	IF		100.0	100.0	A
pB27_A-285	5p/3p	Homo sapiens - DYNLRB1	-175..236	IF		100.0	99.3	A
pB27_A-194	5p/3p	Homo sapiens - DYNLRB1	-175..236	IF		100.0	99.8	A
pB27_A-54	5p/3p	Homo sapiens - DYNLRB1	-175..236	IF		100.0	99.8	A
pB27_A-94	5p/3p	Homo sapiens - DYNLRB1	-175..236	IF		100.0	99.8	A
pB27_A-51	5p/3p	Homo sapiens - DYNLRB1	-175..236	IF		100.0	100.0	A
pB27_A-53	5p/3p	Homo sapiens - DYNLRB1	-175..236	IF		99.5	98.8	A
pB27_A-133	5p/3p	Homo sapiens - DYNLRB1	-175..236	IF		100.0	100.0	A
pB27_A-184	5p/3p	Homo sapiens - DYNLRB1	-175..236	IF		100.0	100.0	A
pB27_A-99	5p/3p	Homo sapiens - DYNLRB1	-175..236	IF		100.0	100.0	A
pB27_A-113	5p/3p	Homo sapiens - DYNLRB1	-175..236	IF		100.0	99.8	A
pB27_A-37	5p/3p	Homo sapiens - DYNLRB1	-175..236	IF		100.0	99.8	A
pB27_A-6	5p/3p	Homo sapiens - DYNLRB1	-175..236	IF		100.0	100.0	A
pB27_A-176	5p/3p	Homo sapiens - DYNLRB1	-145..375	IF		100.0	99.8	A
pB27_A-129	5p	Homo sapiens - DYNLRB1	-145..235	IF		100.0		A
pB27_A-117	5p	Homo sapiens - DYNLRB1	-145..235	IF		99.7		A
pB27_A-52	5p/3p	Homo sapiens - DYNLRB1	-145..236	IF		94.0	100.0	A
pB27_A-254	5p/3p	Homo sapiens - DYNLRB1	-127..234	IF		100.0	100.0	A
pB27_A-267	5p/3p	Homo sapiens - DYNLRB1	-106..344	IF		100.0	100.0	A
pB27_A-274	5p/3p	Homo sapiens - DYNLRB1	-106..238	IF		100.0	99.7	A
pB27_A-250	5p/3p	Homo sapiens - DYNLRB1	-106..235	IF		100.0	100.0	A
pB27_A-218	5p/3p	Homo sapiens - DYNLRB1	-106..235	IF		100.0	100.0	A

Clone Name	Type Seq	Gene Name (Best Match)	Start..Stop (nt)	Frame	Sense	%Id 5p	%Id 3p	PBS
pB27_A-127	5p/3p	Homo sapiens - DYNLRB1	-91..342	IF		99.8	99.5	A
pB27_A-229	5p/3p	Homo sapiens - DYNLRB1	-91..342	IF		99.8	99.8	A
pB27_A-183	5p/3p	Homo sapiens - DYNLRB1	-91..342	IF		99.8	99.5	A
pB27_A-112	5p/3p	Homo sapiens - DYNLRB1	-91..342	IF		99.8	99.8	A
pB27_A-77	5p/3p	Homo sapiens - DYNLRB1	-91..342	IF		99.8	99.5	A
pB27_A-16	5p/3p	Homo sapiens - DYNLRB1	-91..342	IF		99.8	99.8	A
pB27_A-32	5p/3p	Homo sapiens - EIF3C	1320..1811	IF		100.0	100.0	D
pB27_A-172	5p/3p	Homo sapiens - EIF3C	1320..1811	IF		100.0	100.0	D
pB27_A-264	5p/3p	Homo sapiens - EIF3M	78..1168	IF		99.9	99.7	B
pB27_A-209	5p	Homo sapiens - EIF3M	372	IF		97.8		B
pB27_A-282	5p	Homo sapiens - EIF3M	372	IF		97.5		B
pB27_A-132	5p/3p	Homo sapiens - FAM65B	345..838	IF		99.8	99.8	D
pB27_A-63	5p/3p	Homo sapiens - FAM65B	345..838	IF		99.6	98.6	D
pB27_A-306	5p	Homo sapiens - FAM65B	345..839	IF		99.6		D
pB27_A-173	5p/3p	Homo sapiens - FAM65B	345..838	IF		99.8	99.6	D
pB27_A-283	5p/3p	Homo sapiens - FAM65B	345..838	IF		99.0	99.8	D
pB27_A-310	5p/3p	Homo sapiens - HIPK1	2412..3008	IF		100.0	99.5	E
pB27_A-232	5p/3p	Homo sapiens - HIPK1	2598..3303	IF		99.7	99.8	E
pB27_A-197	5p/3p	Homo sapiens - HIPK3 var1	2559..3964	IF		99.7	99.8	D
pB27_A-261	5p/3p	Homo sapiens - HIPK3 var1	2559..3964	IF		93.5	100.0	D
pB27_A-125	5p/3p	Homo sapiens - IMMT	510..1124	IF		100.0	99.0	D
pB27_A-66	5p/3p	Homo sapiens - IMMT	510..1124	IF		100.0	92.8	D
pB27_A-161	5p	Homo sapiens - IMMT	510	IF		98.7		D
pB27_A-157	5p/3p	Homo sapiens - IMMT	510..1124	IF		99.4	93.2	D
pB27_A-268	5p/3p	Homo sapiens - IMMT	510..1124	IF		100.0	99.4	D
pB27_A-62	3p	Homo sapiens - IQGAP1	..4097	??			99.4	A
pB27_A-304	5p/3p	Homo sapiens - IQGAP1	2541..4097	IF		99.8	99.8	A
pB27_A-121	5p/3p	Homo sapiens - IQGAP1	2541..4097	IF		99.4	99.5	A
pB27_A-179	5p/3p	Homo sapiens - IQGAP1	2547..4097	IF		99.8	99.7	A
pB27_A-31	5p/3p	Homo sapiens - IQGAP1	2547..4097	IF		99.6	99.4	A
pB27_A-151	5p/3p	Homo sapiens - IQGAP1	2625..4232	IF		98.2	98.2	A
pB27_A-93	5p/3p	Homo sapiens - IQGAP1	2625..4232	IF		99.6	99.0	A
pB27_A-273	5p/3p	Homo sapiens - IQGAP1	2889..4214	IF		100.0	100.0	A
pB27_A-269	5p/3p	Homo sapiens - IQGAP1	2889..4214	IF		99.7	99.8	A
pB27_A-193	5p/3p	Homo sapiens - IQGAP1	2889..4214	IF		99.6	99.5	A
pB27_A-174	5p/3p	Homo sapiens - IQGAP1	2889..4214	IF		99.7	99.5	A
pB27_A-104	5p/3p	Homo sapiens - NRBP1	825..1682	IF		99.7	99.8	A
pB27_A-216	5p/3p	Homo sapiens - NRBP1	840..1683	IF		99.7	99.4	A
pB27_A-4	5p/3p	Homo sapiens - NRBP1	840..1683	IF		99.7	98.9	A
pB27_A-166	5p/3p	Homo sapiens - NRBP1	840..1683	IF		90.7	98.0	A
pB27_A-39	5p/3p	Homo sapiens - NRBP1	840..1683	IF		99.3	99.1	A
pB27_A-154	5p/3p	Homo sapiens - NRBP1	840..1683	IF		95.7	92.8	A
pB27_A-50	5p/3p	Homo sapiens - NRBP1	840..1683	IF		99.7	98.4	A
pB27_A-9	5p/3p	Homo sapiens - NRBP1	849..1680	IF		99.9	99.2	A
pB27_A-253	5p/3p	Homo sapiens - NRBP1	849..1680	IF		96.2	98.2	A
pB27_A-210	5p/3p	Homo sapiens - NRBP1	849..1680	IF		99.1	99.2	A
pB27_A-57	5p/3p	Homo sapiens - NRBP1	849..1680	IF		99.9	99.1	A
pB27_A-45	5p/3p	Homo sapiens - NRBP1	849..1680	IF		97.5	95.9	A
pB27_A-35	5p/3p	Homo sapiens - NRBP1	855..1950	IF		99.7	98.5	A
pB27_A-101	5p/3p	Homo sapiens - NRBP1	855..1950	IF		99.9	99.5	A
pB27_A-23	5p/3p	Homo sapiens - NRBP1	861..1709	IF		100.0	100.0	A

HYBRIGENICS CONFIDENTIAL - 1104538 - M. Konkel

Clone Name	Type Seq	Gene Name (Best Match)	Start..Stop (nt)		Frame	Sense	%Id 5p	%Id 3p	PBS
pB27_A-180	5p/3p	Homo sapiens - NRBP1	861..1709	X	IF		99.5	99.8	A
pB27_A-182	5p/3p	Homo sapiens - NRBP1	861..1709	X	IF		100.0	99.8	A
pB27_A-41	5p/3p	Homo sapiens - NRBP1	873..1590		IF		97.8	94.1	A
pB27_A-28	5p/3p	Homo sapiens - NRBP1	912..1685	X	IF		99.9	99.7	A
pB27_A-196	5p/3p	Homo sapiens - NRBP1	912..1685	X	IF		100.0	99.5	A
pB27_A-135	5p/3p	Homo sapiens - NRBP1	912..1685	X	IF		96.9	95.4	A
pB27_A-303	5p/3p	Homo sapiens - NRBP1	912..1685	X	IF		99.7	99.3	A
pB27_A-85	5p/3p	Homo sapiens - NRBP1	912..1685	X	IF		99.8	99.3	A
pB27_A-3	5p/3p	Homo sapiens - NRBP1	924..1708	X	IF		99.6	99.5	A
pB27_A-21	5p/3p	Homo sapiens - NRBP1	924..1708	X	IF		98.9	96.0	A
pB27_A-17	5p/3p	Homo sapiens - NRBP1	924..1708	X	IF		98.8	99.2	A
pB27_A-14	5p/3p	Homo sapiens - NRBP1	924..1708	X	IF		99.9	99.8	A
pB27_A-291	5p/3p	Homo sapiens - NRBP1	924..1708	X	IF		100.0	99.7	A
pB27_A-152	5p/3p	Homo sapiens - NRBP1	924..1708	X	IF		97.8	98.3	A
pB27_A-156	5p/3p	Homo sapiens - NRBP1	924..1708	X	IF		99.7	99.8	A
pB27_A-102	5p/3p	Homo sapiens - NRBP1	936..1627	X	IF		99.3	99.3	A
pB27_A-128	5p/3p	Homo sapiens - NRBP1	936..1627	X	IF		97.2	98.5	A
pB27_A-95	5p/3p	Homo sapiens - NRBP1	966..1680	X	IF		99.7	99.7	A
pB27_A-149	5p/3p	Homo sapiens - NRBP1	966..1680	X	IF		92.3	94.1	A
pB27_A-307	5p/3p	Homo sapiens - NRBP1	966..1680	X	IF		100.0	100.0	A
pB27_A-110	5p/3p	Homo sapiens - NRBP1	972..1710	X	IF		100.0	99.5	A
pB27_A-88	5p/3p	Homo sapiens - NRBP1	972..1710	X	IF		100.0	99.5	A
pB27_A-8	5p/3p	Homo sapiens - NRBP1	981..1686	X	IF		99.9	99.5	A
pB27_A-246	5p/3p	Homo sapiens - NRBP1	981..1587		IF		99.0	99.7	A
pB27_A-169	5p/3p	Homo sapiens - NRBP1	981..1686	X	IF		99.9	99.7	A
pB27_A-170	5p/3p	Homo sapiens - NRBP1	981..1686	X	IF		100.0	99.7	A
pB27_A-186	5p/3p	Homo sapiens - NRBP1	981..1687	X	IF		99.4	98.6	A
pB27_A-19	5p/3p	Homo sapiens - NRBP1	981..1686	X	IF		100.0	99.7	A
pB27_A-18	5p/3p	Homo sapiens - NRBP1	981..1686	X	IF		100.0	99.7	A
pB27_A-26	5p/3p	Homo sapiens - NRBP1	981..1587		IF		99.8	99.7	A
pB27_A-192	5p/3p	Homo sapiens - NRBP1	981..1587		IF		100.0	99.8	A
pB27_A-204	5p/3p	Homo sapiens - NRBP1	981..1710	X	IF		100.0	99.5	A
pB27_A-208	5p/3p	Homo sapiens - NRBP1	981..1710	X	IF		100.0	99.2	A
pB27_A-215	5p/3p	Homo sapiens - NRBP1	981..1587		IF		99.7	98.7	A
pB27_A-111	5p/3p	Homo sapiens - NRBP1	981..1686	X	IF		99.7	98.2	A
pB27_A-123	5p/3p	Homo sapiens - NRBP1	981..1687	X	IF		100.0	99.5	A
pB27_A-131	5p/3p	Homo sapiens - NRBP1	981..1587		IF		99.8	99.5	A
pB27_A-139	5p/3p	Homo sapiens - NRBP1	981..1682	X	IF		97.7	97.6	A
pB27_A-55	5p/3p	Homo sapiens - NRBP1	981..1587		IF		91.1	96.2	A
pB27_A-56	5p/3p	Homo sapiens - NRBP1	981..1686	X	IF		99.0	99.1	A
pB27_A-43	5p/3p	Homo sapiens - NRBP1	981..1686	X	IF		99.9	99.5	A
pB27_A-72	5p/3p	Homo sapiens - NRBP1	981..1587		IF		100.0	100.0	A
pB27_A-81	5p/3p	Homo sapiens - NRBP1	981..1682	X	IF		99.6	99.4	A
pB27_A-100	5p/3p	Homo sapiens - NRBP1	981..1710	X	IF		100.0	99.7	A
pB27_A-92	5p/3p	Homo sapiens - NRBP1	993..1682	X	IF		99.8	99.0	A
pB27_A-191	5p/3p	Homo sapiens - NRBP1	993..1682	X	IF		100.0	99.8	A
pB27_A-202	5p/3p	Homo sapiens - NRBP1	993..1682	X	IF		100.0	99.8	A
pB27_A-281	5p/3p	Homo sapiens - NRBP1	993..1682	X	IF		99.0	99.5	A
pB27_A-162	5p/3p	Homo sapiens - NRBP1	993..1682	X	IF		96.0	96.0	A
pB27_A-301	5p/3p	Homo sapiens - NRBP1	993..1682	X	IF		99.4	99.2	A
pB27_A-82	5p/3p	Homo sapiens - NRBP1	993..1682	X	IF		95.3	91.8	A

Clone Name	Type Seq	Gene Name (Best Match)	Start..Stop (nt)		Frame	Sense	%Id 5p	%Id 3p	PBS
pB27_A-188	5p/3p	Homo sapiens - NRBP1	1017..1682	X	IF		99.8	99.0	A
pB27_A-140	5p/3p	Homo sapiens - NRBP1	1026..1697	X	IF		95.6	99.2	A
pB27_A-221	5p/3p	Homo sapiens - NRBP1	1038..1912	X	IF		99.4	98.9	A
pB27_A-255	5p/3p	Homo sapiens - NRBP1	1038..1912	X	IF		99.9	98.1	A
pB27_A-167	5p/3p	Homo sapiens - NRBP1	1038..1912	X	IF		100.0	100.0	A
pB27_A-292	5p	Homo sapiens - NRBP1	1038		IF		99.1		A
pB27_A-241	5p/3p	Homo sapiens - NRBP1	1038..1912	X	IF		98.5	97.2	A
pB27_A-228	5p/3p	Homo sapiens - NRBP1	1038..1912	X	IF		99.9	97.0	A
pB27_A-90	5p/3p	Homo sapiens - NRBP1	1047..1686	X	IF		99.7	98.4	A
pB27_A-212	5p/3p	Homo sapiens - NRBP1	1047..1686	X	IF		99.8	100.0	A
pB27_A-240	5p/3p	Homo sapiens - NRBP1	1047..1686	X	IF		98.3	99.7	A
pB27_A-231	5p/3p	Homo sapiens - NRBP1	1047..1569		IF		100.0	98.9	A
pB27_A-233	5p/3p	Homo sapiens - NRBP1	1047..1569		IF		100.0	100.0	A
pB27_A-64	5p/3p	Homo sapiens - NRBP1	1047..1569		IF		100.0	100.0	A
pB27_A-76	5p/3p	Homo sapiens - NRBP1	1047..1686	X	IF		100.0	99.7	A
pB27_A-287	5p/3p	Homo sapiens - NRBP1	1050..1822	X	IF		100.0	99.8	A
pB27_A-115	5p/3p	Homo sapiens - NRBP1	1050..1822	X	IF		99.5	98.4	A
pB27_A-286	5p/3p	Homo sapiens - NRBP1	1050..1822	X	IF		100.0	99.5	A
pB27_A-60	5p/3p	Homo sapiens - NRBP1	1050..1822	X	IF		100.0	99.2	A
pB27_A-74	5p/3p	Homo sapiens - NRBP1	1050..1822	X	IF		100.0	100.0	A
pB27_A-222	5p/3p	Homo sapiens - NRBP1	1053..1661	X	IF		97.5	98.0	A
pB27_A-258	5p	Homo sapiens - NRBP1	1053		IF		98.1		A
pB27_A-187	5p/3p	Homo sapiens - NRBP1	1053..1661	X	IF		99.8	99.2	A
pB27_A-15	5p/3p	Homo sapiens - NRBP1	1053..1661	X	IF		100.0	99.8	A
pB27_A-119	5p/3p	Homo sapiens - NRBP1	1053..1661	X	IF		100.0	98.4	A
pB27_A-288	5p/3p	Homo sapiens - NRBP1	1053..1843	X	IF		99.9	99.0	A
pB27_A-201	5p/3p	Homo sapiens - NRBP1	1059..1587		IF		100.0	100.0	A
pB27_A-313	5p/3p	Homo sapiens - NRBP1	1059..1587		IF		100.0	100.0	A
pB27_A-237	5p/3p	Homo sapiens - NRBP1	1059..1587		IF		100.0	100.0	A
pB27_A-270	5p/3p	Homo sapiens - NRBP1	1062..1683	X	IF		99.8	99.0	A
pB27_A-220	5p/3p	Homo sapiens - NRBP1	1071..1682	X	IF		100.0	99.7	A
pB27_A-256	5p/3p	Homo sapiens - NRBP1	1071..1786	X	IF		96.7	76.3	A
pB27_A-230	5p/3p	Homo sapiens - NRBP1	1071..1779	X	IF		96.0	95.4	A
pB27_A-244	5p/3p	Homo sapiens - NRBP1	1071..1682	X	IF		92.6	93.8	A
pB27_A-243	5p/3p	Homo sapiens - NRBP1	1071..1786	X	IF		84.8	90.3	A
pB27_A-168	5p/3p	Homo sapiens - NRBP1	1071..1779	X	IF		99.8	99.8	A
pB27_A-195	5p/3p	Homo sapiens - NRBP1	1071..1682	X	IF		100.0	99.7	A
pB27_A-108	5p	Homo sapiens - NRBP1	1071		IF		97.2		A
pB27_A-293	5p/3p	Homo sapiens - NRBP1	1071..1682	X	IF		99.8	98.5	A
pB27_A-295	5p/3p	Homo sapiens - NRBP1	1071..1779	X	IF		99.4	96.9	A
pB27_A-296	5p/3p	Homo sapiens - NRBP1	1071..1779	X	IF		99.8	98.7	A
pB27_A-122	5p/3p	Homo sapiens - NRBP1	1071..1682	X	IF		100.0	99.8	A
pB27_A-130	5p/3p	Homo sapiens - NRBP1	1071..1779	X	IF		99.5	94.3	A
pB27_A-138	5p/3p	Homo sapiens - NRBP1	1071..1586		IF		100.0	100.0	A
pB27_A-289	5p/3p	Homo sapiens - NRBP1	1071..1779	X	IF		99.9	99.5	A
pB27_A-312	5p/3p	Homo sapiens - NRBP1	1071..1779	X	IF		100.0	99.3	A
pB27_A-142	5p/3p	Homo sapiens - NRBP1	1071..1683	X	IF		99.8	99.8	A
pB27_A-163	5p	Homo sapiens - NRBP1	1071		IF		99.0		A
pB27_A-71	5p/3p	Homo sapiens - NRBP1	1071..1586		IF		97.0	99.0	A
pB27_A-97	5p/3p	Homo sapiens - NRBP1	1071..1779	X	IF		100.0	99.7	A
pB27_A-265	5p/3p	Homo sapiens - NRBP1	1080..1777	X	IF		100.0	99.5	A

HYBRIGENICS CONFIDENTIAL - 1104538 - M. Konkel

Clone Name	Type Seq	Gene Name (Best Match)	Start..Stop (nt)	Frame	Sense	%Id 5p	%Id 3p	PBS
pB27_A-160	5p/3p	Homo sapiens - NRBP1	1080..1777 ✕	IF		99.6	99.4	A
pB27_A-80	5p/3p	Homo sapiens - NRBP1	1080..1777 ✕	IF		100.0	99.0	A
pB27_A-12	5p/3p	Homo sapiens - NRBP1	1098..1961 ✕	IF		99.8	99.2	A
pB27_A-190	5p/3p	Homo sapiens - NRBP1	1098..1961 ✕	IF		99.7	99.7	A
pB27_A-214	5p/3p	Homo sapiens - NRBP1	1098..1961 ✕	IF		99.4	97.0	A
pB27_A-103	5p/3p	Homo sapiens - NRBP1	1098..1961 ✕	IF		99.7	98.4	A
pB27_A-200	5p/3p	Homo sapiens - NRBP1	1101..1681 ✕	IF		99.8	99.5	A
pB27_A-259	5p/3p	Homo sapiens - NRBP1	1110..1591	IF		100.0	100.0	A
pB27_A-227	5p/3p	Homo sapiens - NRBP1	1122..1683 ✕	IF		100.0	99.0	A
pB27_A-219	5p/3p	Homo sapiens - NRBP1	1128..1772 ✕	IF		99.8	99.7	A
pB27_A-251	5p/3p	Homo sapiens - NRBP1	1128..1772 ✕	IF		99.7	99.5	A
pB27_A-33	5p/3p	Homo sapiens - NRBP1	1128..1772 ✕	IF		97.4	97.3	A
pB27_A-271	5p/3p	Homo sapiens - NRBP1	1128..1772 ✕	IF		99.7	99.5	A
pB27_A-309	5p/3p	Homo sapiens - NRBP1	1128..1772 ✕	IF		100.0	99.8	A
pB27_A-235	5p/3p	Homo sapiens - NRBP1	1128..1772 ✕	IF		94.8	98.6	A
pB27_A-126	5p/3p	Homo sapiens - NRBP1	1191..1921 ✕	IF		99.7	98.8	A
pB27_A-68	5p	Homo sapiens - NRBP1	1191	IF		97.9		A
pB27_A-272	5p/3p	Homo sapiens - NRBP1	1218..1624 ✕	IF		90.6	99.5	A
pB27_A-29	5p/3p	Homo sapiens - NRBP1	1248..1825 ✕	IF		100.0	100.0	A
pB27_A-189	5p/3p	Homo sapiens - NRBP1	1248..1825 ✕	IF		99.7	99.1	A
pB27_A-109	5p/3p	Homo sapiens - NRBP1	1254..1668 ✕	IF		100.0	100.0	A
pB27_A-262	5p/3p	Homo sapiens - NRBP1	1257..1687 ✕	IF		100.0	100.0	A
pB27_A-5	5p/3p	Homo sapiens - NRBP1	1257..1687 ✕	IF		100.0	100.0	A
pB27_A-249	5p/3p	Homo sapiens - NRBP1	1257..1687 ✕	IF		96.3	100.0	A
pB27_A-24	5p/3p	Homo sapiens - NRBP1	1257..1687 ✕	IF		100.0	100.0	A
pB27_A-205	5p/3p	Homo sapiens - NRBP1	1257..1687 ✕	IF		100.0	100.0	A
pB27_A-145	5p/3p	Homo sapiens - NRBP1	1257..1687 ✕	IF		100.0	100.0	A
pB27_A-1	5p/3p	Homo sapiens - NRBP1	1266..1669 ✕	IF		100.0	100.0	A
pB27_A-185	5p/3p	Homo sapiens - NRBP1	1266..1669 ✕	IF		100.0	100.0	A
pB27_A-207	5p/3p	Homo sapiens - NRBP1	1266..1669 ✕	IF		100.0	100.0	A
pB27_A-279	5p/3p	Homo sapiens - NRBP1	1284..1931 ✕	IF		99.7	99.4	A
pB27_A-266	5p/3p	Homo sapiens - NRBP1	1287..1680 ✕	IF		100.0	100.0	A
pB27_A-91	5p/3p	Homo sapiens - NRBP1	1287..1682 ✕	IF		100.0	100.0	A
pB27_A-20	5p/3p	Homo sapiens - NRBP1	1287..1680 ✕	IF		100.0	100.0	A
pB27_A-40	5p/3p	Homo sapiens - NRBP1	1287..1680 ✕	IF		100.0	99.7	A
pB27_A-120	5p/3p	Homo sapiens - NRBP1	1287..1682 ✕	IF		100.0	100.0	A
pB27_A-146	5p/3p	Homo sapiens - NRBP1	1287..1708 ✕	IF		100.0	100.0	A
pB27_A-59	5p/3p	Homo sapiens - NRBP1	1287..1680 ✕	IF		99.2	100.0	A
pB27_A-61	5p/3p	Homo sapiens - NRBP1	1287..1682 ✕	IF		100.0	100.0	A
pB27_A-177	5p	Homo sapiens - NRBP1	1317	IF		95.9		A
pB27_A-226	5p/3p	Homo sapiens - NRBP1	1356..1945 ✕	IF		99.8	98.5	A
pB27_A-278	5p/3p	Homo sapiens - NRBP1	1356..1945 ✕	IF		100.0	99.5	A
pB27_A-148	5p/3p	Homo sapiens - NRBP1	1356..1945 ✕	IF		91.6	92.9	A
pB27_A-46	5p/3p	Homo sapiens - NRBP1	1356..1945 ✕	IF		100.0	99.0	A
pB27_A-223	5p/3p	Homo sapiens - NRBP1	1362..1920 ✕	IF		99.5	98.1	A
pB27_A-217	5p/3p	Homo sapiens - NRBP1	1362..1920 ✕	IF		100.0	99.1	A
pB27_A-260	5p/3p	Homo sapiens - NRBP1	1362..1920 ✕	IF		97.2	93.4	A
pB27_A-248	5p/3p	Homo sapiens - NRBP1	1362..1920 ✕	IF		98.9	98.1	A
pB27_A-58	5p/3p	Homo sapiens - NRBP1	1362..1920 ✕	IF		98.9	95.0	A
pB27_A-11	5p/3p	Homo sapiens - NRBP1	1362..1920 ✕	IF		100.0	99.5	A
pB27_A-10	5p/3p	Homo sapiens - NRBP1	1362..1920 ✕	IF		100.0	99.5	A

HYBRIGENICS SERVICES

Clone Name	Type Seq	Gene Name (Best Match)	Start..Stop (nt)		Frame	Sense	%Id 5p	%Id 3p	PBS
pB27_A-242	5p/3p	Homo sapiens - NRBP1	1362..1920	X	IF		96.8	84.9	A
pB27_A-178	5p	Homo sapiens - NRBP1	1362		IF		95.6		A
pB27_A-181	5p/3p	Homo sapiens - NRBP1	1362..1920	X	IF		100.0	99.6	A
pB27_A-206	5p/3p	Homo sapiens - NRBP1	1362..1920	X	IF		100.0	99.3	A
pB27_A-106	5p/3p	Homo sapiens - NRBP1	1362..1920	X	IF		100.0	99.6	A
pB27_A-136	5p/3p	Homo sapiens - NRBP1	1362..1920	X	IF		98.6	97.2	A
pB27_A-284	5p/3p	Homo sapiens - NRBP1	1362..1920	X	IF		99.8	98.6	A
pB27_A-276	5p/3p	Homo sapiens - NRBP1	1362..1920	X	IF		100.0	99.5	A
pB27_A-65	5p/3p	Homo sapiens - NRBP1	1362..1920	X	IF		100.0	99.5	A
pB27_A-211	5p/3p	Homo sapiens - NRBP1	1368..1930	X	IF		98.9	98.9	A
pB27_A-44	5p/3p	Homo sapiens - NRBP1	1368..1930	X	IF		99.8	97.0	A
pB27_A-275	5p/3p	Homo sapiens - NRBP1	1368..1930	X	IF		99.8	98.1	A
pB27_A-73	5p/3p	Homo sapiens - NRBP1	1368..1930	X	IF		99.8	99.5	A
pB27_A-175	5p	Homo sapiens - PCDH1	385		OOF1		99.4		N/A
pB27_A-164	5p	Homo sapiens - PCDH1	385		OOF1		99.7		N/A
pB27_A-134	5p/3p	Homo sapiens - PIAS1	1020..1511		IF		100.0	99.8	E
pB27_A-30	5p/3p	Homo sapiens - PIAS1	1020..1511		IF		100.0	100.0	E
pB27_A-107	5p/3p	Homo sapiens - PIAS3	918..1395		IF		100.0	100.0	E
pB27_A-302	5p/3p	Homo sapiens - RNF111	315..1554		IF		98.3	100.0	D
pB27_A-203	5p/3p	Homo sapiens - SEMA7A	1792..1087		??	N	99.4	99.3	N/A
pB27_A-116	5p/3p	Homo sapiens - SEMA7A	1792..1087		??	N	97.3	98.0	N/A
pB27_A-70	5p/3p	Homo sapiens - SEMA7A	1792..1087		??	N	89.1	90.5	N/A
pB27_A-257	5p/3p	Homo sapiens - SEMA7A	1798..626		??	N	96.0	83.1	N/A
pB27_A-245	5p/3p	Homo sapiens - SEMA7A	1798..626		??	N	94.0	94.9	N/A
pB27_A-280	5p/3p	Homo sapiens - SEMA7A	1798..626		??	N	97.3	97.4	N/A
pB27_A-141	5p/3p	Homo sapiens - SEMA7A	1798..626		??	N	99.5	99.5	N/A
pB27_A-137	5p/3p	Homo sapiens - SEMA7A	1798..626		??	N	99.4	99.1	N/A
pB27_A-159	5p/3p	Homo sapiens - SEMA7A	1798..626		??	N	99.4	99.2	N/A
pB27_A-114	5p/3p	Homo sapiens - SEMA7A	1798..1109		??	N	98.8	98.0	N/A
pB27_A-86	5p/3p	Homo sapiens - SEMA7A	1798..1109		??	N	99.7	98.6	N/A
pB27_A-118	5p/3p	Homo sapiens - SEMA7A	1798..626		??	N	99.7	99.5	N/A
pB27_A-13	5p/3p	Homo sapiens - SEMA7A	1798..626		??	N	99.7	99.2	N/A
pB27_A-7	5p/3p	Homo sapiens - SEMA7A	1798..626		??	N	98.8	98.7	N/A
pB27_A-84	5p/3p	Homo sapiens - SEMA7A	1798..626		??	N	99.7	99.1	N/A
pB27_A-150	5p/3p	Homo sapiens - SNRNP70 var2	36..626		IF		94.1	93.0	F
pB27_A-314	5p/3p	Homo sapiens - SNRNP70 var2	36..626		IF		97.1	96.8	F
pB27_A-305	5p/3p	Homo sapiens - SNRNP70 var2	36..625		IF		97.1	86.2	F
pB27_A-155	5p/3p	Homo sapiens - TDG	210..1101		IF		100.0	99.7	E
pB27_A-213	5p/3p	Homo sapiens - TDG	210..1101		IF		100.0	99.8	E
pB27_A-98	5p/3p	Homo sapiens - TDG	228..2468	X	IF		98.2	99.2	E
pB27_A-96	5p/3p	Homo sapiens - UBE2I variant 1	-106..353		IF		100.0	99.1	E
pB27_A-83	5p/3p	Homo sapiens - UBE2I variant 1	-106..353		IF		97.6	95.0	E
pB27_A-124	5p/3p	Homo sapiens - UBE2I variant 1	-106..353		IF		100.0	100.0	E
pB27_A-47	5p/3p	Homo sapiens - UBE2I variant 1	-106..353		IF		100.0	100.0	E
pB27_A-263	5p/3p	Homo sapiens - ZMYM2	633..4922	X	IF		100.0	58.6	E
pB27_A-199	5p/3p	Homo sapiens - ZMYM2	633..4922	X	IF		99.7	58.2	E
pB27_A-224	5p/3p	Homo sapiens - ZMYM2	906..1793		IF		99.5	98.7	E
pB27_A-252	5p/3p	Homo sapiens - ZMYM2	924..1790		IF		97.5	94.6	E
pB27_A-147	5p/3p	Homo sapiens - ZNF106	3762..4354		IF		92.3	94.7	D
pB27_A-290	5p/3p	Homo sapiens - GenMatch	-1..463	X	IF		85.3	76.0	D
pB27_A-297	5p/3p	Homo sapiens - GenMatch	-1..839	X	IF		99.9	58.5	D

Clone Name	Type Seq	Gene Name (Best Match)	Start..Stop (nt)	Frame	Sense	%Id 5p	%Id 3p	PBS
pB27_A-247	5p/3p	Homo sapiens - GenMatch	-1..631 ✕	IF		99.0	98.9	D

HYBRiGENiCS SERVICES

DomSight: PLA_RP6_hgx4788v1 vs. Human Placenta_RP6 (30 Jun 2017)

(Bait plasmid(s): hgx4788v1_pB27)

Bait fragment
SID fragment
Pfam or SMART domain
Transmembrane domain (TMHMM)
Coiled-coil domain (Ncoils)
Signal peptide (SignalP)

SID: Selected Interaction Domain
It is the amino acid sequence shared by all prey fragments matching the same reference protein. SIDs have been found in numerous cases to correspond to an identified structural or functional domain.
Protein Display:
Proteins larger than 1500 aa are shown in sections of 1499 aa each. Only sections containing bait fragments, SIDs or predicted functional and structural domains are represented.

Legend

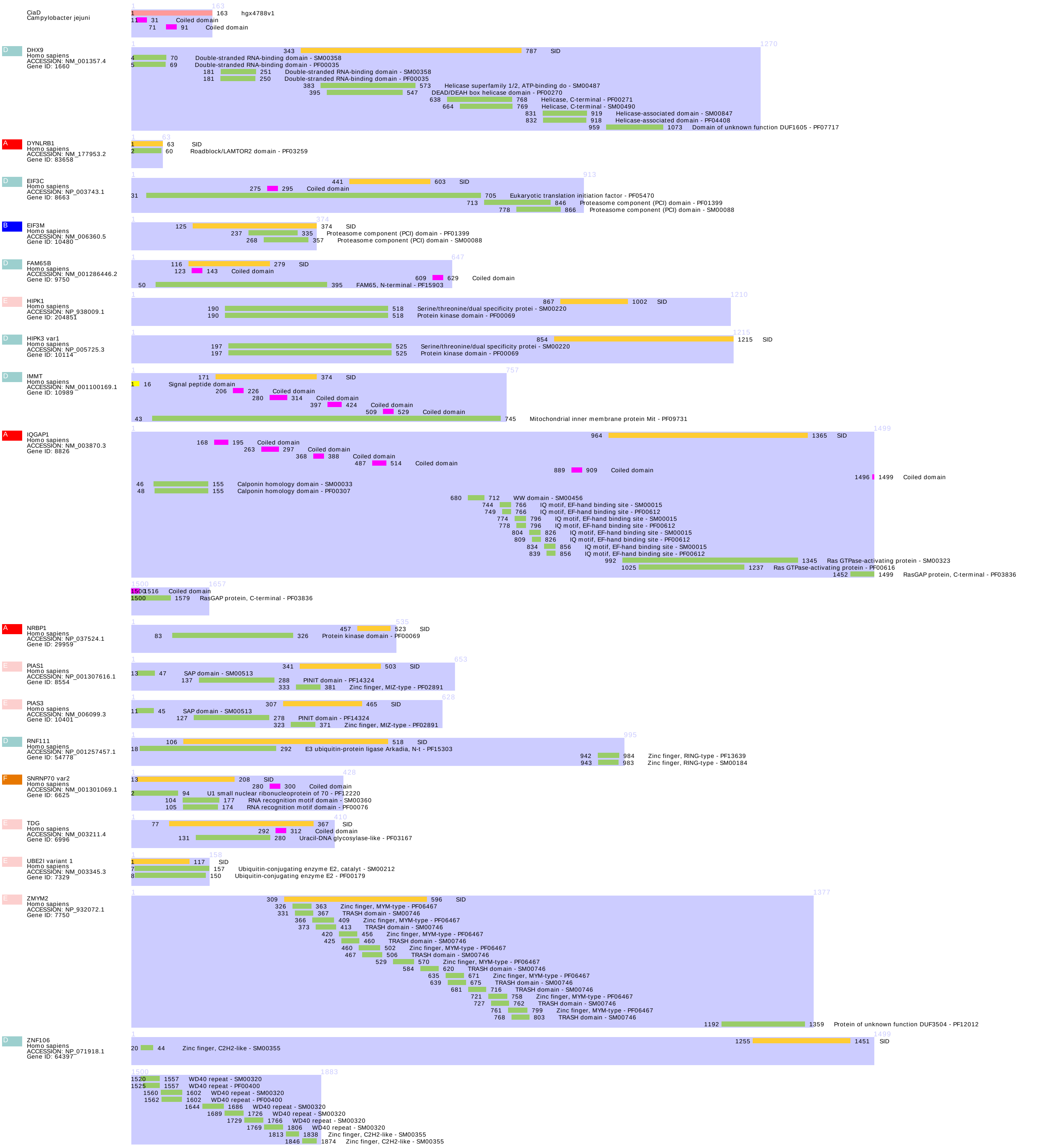

DomSight: PLA_RP6_hgx4788v1 vs. Human Placenta_RP6 (30 Jun 2017)

(Bait plasmid(s): hgx4788v1_pB27)

Legend		
	Bait fragment	*SID: Selected Interaction Domain*
	SID fragment	*It is the amino acid sequence shared by all prey fragments matching the same reference protein. SIDs have been found in numerous cases to correspond to an identified structural or functional domain.*
	Pfam or SMART domain	*Protein Display:*
	Transmembrane domain (TMHMM)	*Proteins larger than 1500 aa are shown in sections of 1499 aa each. Only sections containing bait fragments, SIDs or predicted functional and structural domains are represented.*
	Coiled-coil domain (Ncoils)	
	Signal peptide (SignalP)	

Legend

D GenMatch
Homo sapiens
ACCESSION: NG_029628.1

1 229
1 154 SID

D GenMatch
Homo sapiens
ACCESSION: NG_031945.1

1 224
1 210 SID
