# Supplementary material for: The Campylobacter jejuni CiaD effector co-opts the host cell protein IQGAP1 to promote cell entry
Source: Nat Commun. 2021 Feb 26;12:1339. doi: 10.1038/s41467-021-21579-5 (PMC7910587; doi:10.1038/s41467-021-21579-5)
Supplement: Supplementary file 5 — Reporting Summary [file 41467_2021_21579_MOESM5_ESM.pdf]

nature research

Corresponding author(s): Michael E. Konkel

Last updated by author(s): 01/21/2021

Reporting Summary

Nature Research wishes to improve the reproducibility of the work that we publish. This form provides structure for consistency and transparency in reporting. For further information on Nature Research policies, see Authors & Referees and the Editorial Policy Checklist .

Statistics

For all statistical analyses, confirm that the following items are present in the figure legend, table legend, main text, or Methods section.

n/a	Confirmed	
☐	☒	The exact sample size (*n*) for each experimental group/condition, given as a discrete number and unit of measurement
☒	☐	A statement on whether measurements were taken from distinct samples or whether the same sample was measured repeatedly
☐	☒	The statistical test(s) used AND whether they are one- or two-sided<br>*Only common tests should be described solely by name; describe more complex techniques in the Methods section.*
☒	☐	A description of all covariates tested
☒	☐	A description of any assumptions or corrections, such as tests of normality and adjustment for multiple comparisons
☐	☒	A full description of the statistical parameters including central tendency (e.g. means) or other basic estimates (e.g. regression coefficient) AND variation (e.g. standard deviation) or associated estimates of uncertainty (e.g. confidence intervals)
☐	☒	For null hypothesis testing, the test statistic (e.g. *F*, *t*, *r*) with confidence intervals, effect sizes, degrees of freedom and *P* value noted<br>*Give P values as exact values whenever suitable.*
☒	☐	For Bayesian analysis, information on the choice of priors and Markov chain Monte Carlo settings
☒	☐	For hierarchical and complex designs, identification of the appropriate level for tests and full reporting of outcomes
☒	☐	Estimates of effect sizes (e.g. Cohen's *d*, Pearson's *r*), indicating how they were calculated

Our web collection on statistics for biologists contains articles on many of the points above.

Software and code

Policy information about availability of computer code

Data collection	ImageQuant LAS 4000 Control Software version 1.1.0.17 was used to collect data from an ImageQuant LAS 4000 Mini imager. ImageJ 1.52a was used to compile microscopy images.
Data analysis	Statistical analysis and plotting was performed with GraphPad Prism 6.0g.

For manuscripts utilizing custom algorithms or software that are central to the research but not yet described in published literature, software must be made available to editors/reviewers. We strongly encourage code deposition in a community repository (e.g. GitHub). See the Nature Research guidelines for submitting code & software for further information.

Data

Policy information about availability of data

All manuscripts must include a data availability statement. This statement should provide the following information, where applicable:

- Accession codes, unique identifiers, or web links for publicly available datasets
- A list of figures that have associated raw data
- A description of any restrictions on data availability

All data generated or analysed during this study are included in this published article (and its supplementary information files). Proteomic data is available at the MassIVE database under the accession MSV000086662. Other data is available in a Source Data file.

Field-specific reporting

Please select the one below that is the best fit for your research. If you are not sure, read the appropriate sections before making your selection.

☒

Life sciences study design

All studies must disclose on these points even when the disclosure is negative.

Sample size	In the interest of reproducibility, the in vitro experiments were repeated at least three times (biological replicates). Assays typically had at least three technical replicates. Due to the lack of previous work needed to estimate an effect size, no sample size calculation was performed prior to the initiation of the work. For every in vitro experiment, we chose to include sufficient technical replicates to understand the variability of the assay. In the absence of a clear understanding of statistical power, the worst outcome of a sample size too small would be a failure to detect a difference - none of the conclusions are based on the failure to detect a difference.
Data exclusions	No data were excluded.
Replication	All assays were repeated with a minimum of three biological replicates, with successful agreement of the results.
Randomization	This is not applicable to this study. This study relied on bacterial and cell culture models. Clonal batches of human and bacterial cells were used for experimentation. While efforts were made to capture any variability in the experimentation, all samples were ultimately drawn from the same freezer stocks of cells.
Blinding	This is not applicable to this study. All results in this study are from in vitro laboratory experiments. The individuals implementing the experiments were the same individuals analyzing the data from the experiments, blinding reagent stocks is impractical.

Reporting for specific materials, systems and methods

We require information from authors about some types of materials, experimental systems and methods used in many studies. Here, indicate whether each material, system or method listed is relevant to your study. If you are not sure if a list item applies to your research, read the appropriate section before selecting a response.

Materials & experimental systems

n/a	Involved in the study	
☐	☒	Antibodies
☐	☒	Eukaryotic cell lines
☒	☐	Palaeontology
☒	☐	Animals and other organisms
☒	☐	Human research participants
☒	☐	Clinical data

Methods

n/a	Involved in the study	
☒	☐	ChIP-seq
☒	☐	Flow cytometry
☒	☐	MRI-based neuroimaging

Antibodies

Antibodies used	Polyclonal rabbit anti-C. jejuni: Konkel et al., 1993<br>Anti c-Myc mouse monoclonal (Clone 9E10): Takara Bio Cat# 631206<br>AlexaFluor 680 anti-rabbit: Jackson ImmunoResearch Cat# 711-625-152, RRID:AB_2340627<br>AlexaFluor 488 anti-mouse: Jackson ImmunoResearch Cat# 715-545-150, RRID:AB_2340846<br>total-Erk1/2: Santa Cruz Biotechnology Cat# sc-94, RRID:AB_2140110<br>phospho-Erk1/2: Cell Signaling Technology Cat# 9101, RRID:AB_331646<br>IQGAP1: Abcam Cat# ab86064, RRID:AB_1925119<br>Goat anti-rabbit antibody conjugated to horseradish peroxidase: Sigma Cat# A6154, RRID:AB_258284<br>RacGAP1: Thermo Fisher Scientific Cat# PA5-22265, RRID:AB_11155250<br>Rac1/2/3: Cell Signaling Technology Cat# 2465S, RRID:AB_2176152<br>IQGAP1: Santa Cruz Biotechnology Cat# sc-376021, RRID:AB_10988556<br>Anti-mouse IgG-HRP: Sigma Cat# A4416, RRID:AB_258167<br>Anti-FLAG: Sigma Cat# F7425, RRID:AB_439687
Validation	Polyclonal rabbit anti-C. jejuni:<br>Tested in the absence of C. jejuni for non-specific immunofluorescence, no cross reactivity noted.<br><br>Anti c-Myc mouse monoclonal: Takara Bio Cat# 631206<br>In the data presented in the paper, no cross reactivity was noted in cells lacking c-Myc expression. Human INT407 cultured epithelial cells expressing a c-Myc tagged plasmid were stained with an equal concentration of antibody as cells that were not transected. Imaging at the same illumination settings only showed signal in the transfected cells. From the manufacturer: "This antibody was tested by Western blotting. Mammalian cell lysate containing an expressed c-Myc-tagged protein was electrophoresed on an SDS polyacrylamide gel, followed by transfer to a PVDF membrane. The blot was probed with 2 µg/ml c-Myc Monoclonal Antibody, followed by secondary goat anti-mouse antibody conjugated to horseradish peroxidase. The signal was detected using a chemiluminescent detection system."

AlexaFluor 680 anti-rabbit: Jackson ImmunoResearch Cat# 711-625-152, RRID:AB_2340627
From the manufacturer: “Based on immunoelectrophoresis and/or ELISA, the antibody reacts with whole molecule rabbit IgG. It also reacts with the light chains of other rabbit immunoglobulins. No antibody was detected against non-immunoglobulin serum proteins. The antibody has been tested by ELISA and/or solid-phase adsorbed to ensure minimal cross-reaction with bovine, chicken, goat, guinea pig, syrian hamster, horse, human, mouse, rat and sheep serum proteins, but it may cross-react with immunoglobulins from other species.” Furthermore, in laboratory experiments lacking primary antibodies, no fluorescence if noted.

AlexaFluor 488 anti-mouse: Jackson ImmunoResearch Cat# 715-545-150, RRID:AB_2340846
From the manufacturer: “Based on immunoelectrophoresis and/or ELISA, the antibody reacts with whole molecule mouse IgG. It also reacts with the light chains of other mouse immunoglobulins. No antibody was detected against non-immunoglobulin serum proteins. The antibody has been tested by ELISA and/or solid-phase adsorbed to ensure minimal cross-reaction with bovine, chicken, goat, guinea pig, syrian hamster, horse, human, rabbit and sheep serum proteins, but it may cross-react with immunoglobulins from other species.” In laboratory experiments lacking primary antibodies, no fluorescence if noted.
total-Erk1/2: Santa Cruz Biotechnology Cat# sc-94, RRID:AB_2140110
From the manufacturer: “polyclonal affinity purified antibody raised against a peptide mapping within subdomain XI of ERK 1”. Laboratory experiments have shown that the antibody reacts with Erk1 and Erk2 as evidenced by the appearance of bands at 44 and 42 kDa.

phospho-Erk1/2: Cell Signaling Technology Cat# 9101, RRID:AB_331646
From the manufacturer: “Phospho-p44/42 MAPK (Erk1/2) (Thr202/Tyr204) Antibody detects endogenous levels of p44 and p42 MAP Kinase (Erk1 and Erk2) when phosphorylated either individually or dually at Thr202 and Tyr204 of Erk1 (Thr185 and Tyr187 of Erk2). The antibody does not cross-react with the corresponding phosphorylated residues of either JNK/SAPK or p38 MAP Kinase, and does not cross-react with non-phosphorylated Erk1/2.”
IQGAP1: Abcam Cat# ab86064, RRID:AB_1925119
In data presented in this paper, the IQGAP1 reactive band disappears after treatment with IQGAP1-specific shRNAs.
Goat anti-rabbit antibody conjugated to horseradish peroxidase: Sigma Cat# A6154, RRID:AB_258284
From the manufacturer: “Goat Anti-Rabbit IgG (whole molecule)-Peroxidase antibody is immunospecific for rabbit IgG by immunoelectrophoresis against normal serum and rabbit IgG, prior to conjugation.”

RacGAP1: Thermo Fisher Scientific Cat# PA5-22265, RRID:AB_11155250
No validation statement from the manufacturer.

Rac1/2/3: Cell Signaling Technology Cat# 2465S, RRID:AB_2176152
From the manufacturer: “Rac1/2/3 Antibody detects endogenous levels of total Rac1/2/3 proteins. This antibody does not cross-react with other small GTPases.”

IQGAP1: Santa Cruz Biotechnology Cat# sc-376021, RRID:AB_10988556
From the manufacturer: “IQGAP1 (C-9) is a mouse monoclonal antibody specific for an epitope mapping between amino acids 3-37 near the N-terminus of IQGAP1 of human origin.”

Anti-mouse IgG-HRP: Sigma Cat# A4416, RRID:AB_258167
From the manufacturer: "Anti-Mouse IgG (whole molecule)-Peroxidase antibody is specific for mouse IgGs. "

Anti-FLAG: Sigma Cat# F7425, RRID:AB_439687
From the manufacturer: "TThe rabbit Anti-FLAG polyclonal affinity antibody ANTI-FLAG recognizes the FLAG epitope located on FLAG fusion proteins. This antibody reacts with N-terminal, N-terminal-Met, and C-terminal FLAG fusion proteins. Purified by affinity chromatography on a column bearing the immunizing peptide. "

Eukaryotic cell lines

Policy information about cell lines

Cell line source(s)	Human INT 407 cells were initially sourced from ATCC.
Authentication	Cell lines have not been authenticated.
Mycoplasma contamination	Cell lines were routinely stained with a nuclear stain (DAPI) and examined for microbial contamination. No mycoplasma contamination has been observed.
Commonly misidentified lines (See ICLAC register)	The INT 407 cell line is a result of HeLa contamination, and therefore are considered HeLa cells. INT 407 cells were used in this study due to their long history in C. jejuni research, and the driving requirement of this study was the use of a generic human epithelial cell. Specifically, the cultured cells served as a source of human proteins for pulldown and immunoprecipitation experiments, and a cellular host for internalized C. jejuni.
